# Underwater Suit-Wearing Cyborg Insect Capable of Hours-Long Diving and Terra-Aqua Travel

**DOI:** 10.1038/s41467-026-74235-1

**Published:** 2026-06-29

**Authors:** Zifu FAN, Kazuki KAI, Kewei SONG, Duc Long LE, Thu Ha TRAN, Mingyu HAO, Wei Yang WAN, Shinjiro UMEZU, Hirotaka SATO

**Affiliations:** 1 School of Mechanical and Aerospace Engineering, Nanyang Technological University, Singapore, 637460 Singapore; 2 School of Electrical and Electronic Engineering, Nanyang Technological University, Singapore, 639798 Singapore; 3 School of Creative Science and Engineering, Waseda University, Tokyo, 169-8555 Japan

**Keywords:** Mechanical engineering, Electrical and electronic engineering

## Abstract

The fundamental operational range of cyborg insects, which are hybrid robots that combine a living insect with an electronic controller, is inherently restricted to the host’s natural environment. To extend their operational range, we developed a wearable diving suit for terrestrial insects. The suit integrates a miniaturised oxygen generation module with a flexible waterproof shell, enabling continuous oxygen supply and isolation from surrounding water. By fitting a cockroach, which is a terrestrial species, into this diving suit, we allowed it to survive and operate in oxygen-deprived environments such as underwater, transforming it into an amphibious cyborg robot capable of operation across land and water. The suit sustained respiration and locomotion for up to 3 h underwater, establishing amphibious cyborg insects that combine biological adaptability with engineered protection for prolonged exploration in extreme, confined environments.

## Abstract (web summary)

A wearable diving suit enables terrestrial cyborg cockroaches to maintain respiration and locomotion underwater for up to 3 hours. This biohybrid system facilitates sustained exploration through hybrid hazardous terrains, serving as an amphibious robot.

## Introduction

Cyborg insects are hybrid systems that integrate living insects with electronic components^1–5^, combining the biological capabilities of insects with the technological functions of electromechanical devices to remotely induce their movements. Current cyborg insects are envisioned for use in complex tasks such as search-and-rescue missions^6^, pipeline inspection^7^ and object transportation^8^, with cockroach-based ones considered the most promising owing to their robustness and ease of locomotion control. Unlike conventional artificial small robots which consume substantial power to drive actuators, draining the energy stored in their onboard batteries, cyborg insects locomote with the insects’ own muscles, requiring no electrical actuation and achieving minimal power consumption^9,10^. Their compact size, adaptability, and robustness allow them to traverse cluttered environments and enter into confined spaces inaccessible to larger robots^11^.

However, their operation is constrained by the host’s physiological requirements, such as optimal oxygen and temperature levels. Naturally, the inability of terrestrial hosts like cockroaches to absorb aquatic oxygen prevents underwater functions^12^. Given that real-world search-and-rescue or infrastructure inspection terrains often include puddles, flooded zones, or other partially submerged areas, continuous operation requires developing cyborg insects capable of temporary submersion and locomotion underwater while maintaining normal metabolic activity.

**Fig. 1:**
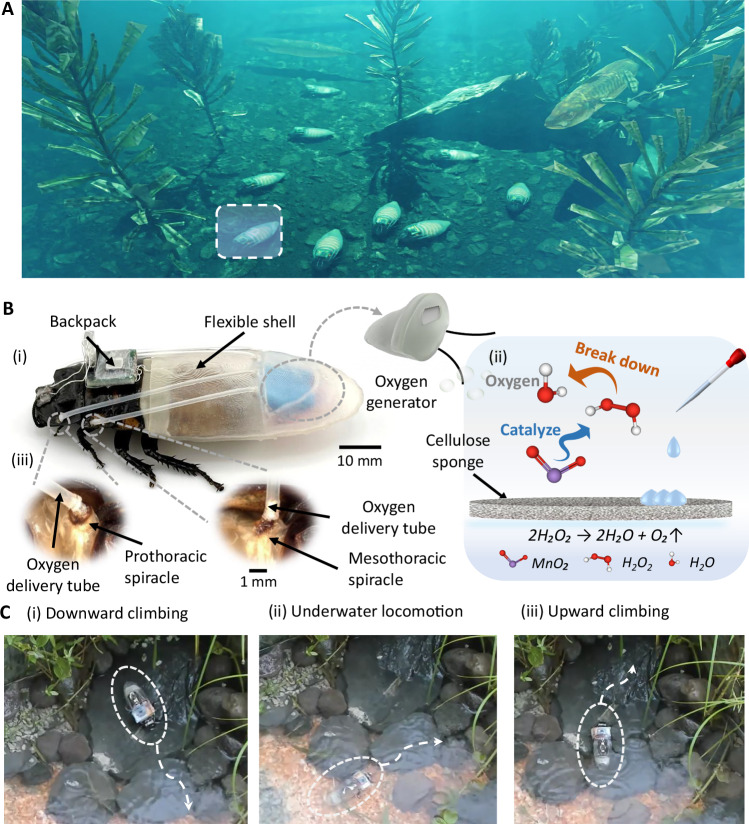
Concept and design of amphibious cyborg insect system. **A** Conceptual illustration of the cyborg insect operating underwater. **B** Structural design and oxygen generation mechanism of diving suit for cyborg cockroaches. i) Diving suit comprises a flexible waterproof shell, an integrated oxygen generator and oxygen delivery tubes. ii) Oxygen generator employs a MnO_2_-H_2_O_2_ catalytic reaction on a cellulose sponge and generates oxygen and water. iii) Generated oxygen is delivered to the prothoracic and mesothoracic spiracles through oxygen delivery tubes. **C** Demonstration of real-world locomotion. Photographs of the cyborg insect with a diving suit performing i) downward climbing, ii) underwater locomotion, and iii) upward climbing.

If a miniature unit capable of supplying oxygen could be mounted onto a cockroach’s body, it might be possible to realise a cyborg cockroach that operates both on land and underwater. Cockroaches, like most terrestrial insects, breathe through thoracic spiracles that take in oxygen directly from the air^13,14^. If oxygen could be supplied to these spiracles while preventing water entry, cyborg insects might be able to operate underwater as well as on land. To realise this concept, we designed a compact and self-contained oxygen supply system, referred to as a ‘diving suit’, based on a controlled chemical reaction that gradually releases oxygen without requiring electronic components. Utilising the Madagascar hissing cockroach (*Gromphadorhina portentosa*) as the biological platform, a wearable diving suit comprising a flexible shell, an oxygen generator and oxygen delivery tubes was designed enabling survival and task execution during prolonged submersion (Fig. 1A). The flexible abdominal shell insulates the abdominal spiracles from surrounding water and acts as an oxygen storage and transport tank (Fig. 1B, i). The oxygen generator is a sealed chamber containing a hydrogen peroxide (H_2_O_2_) solution and a manganese dioxide (MnO_2_) catalyst. Under catalytic action, the H_2_O_2_ decomposes to produce oxygen (Fig. 1B, ii) to maintain the insects’ normal respiratory function. The oxygen delivery tubes connect the flexible shell to the cockroach’s thoracic spiracles (Fig. 1B, iii), transporting the generated oxygen to the tracheae. Together, these components enable cockroaches to achieve amphibious locomotion (Fig. 1C). This study presents an amphibious cyborg insect capable of user-induced locomotion with a low-power, compact design, that enables long-duration operation in confined and cluttered terrestrial–aquatic environments.

## Results and discussion

### Integrative design of diving suit for underwater survival and mobility

**Fig. 2:**
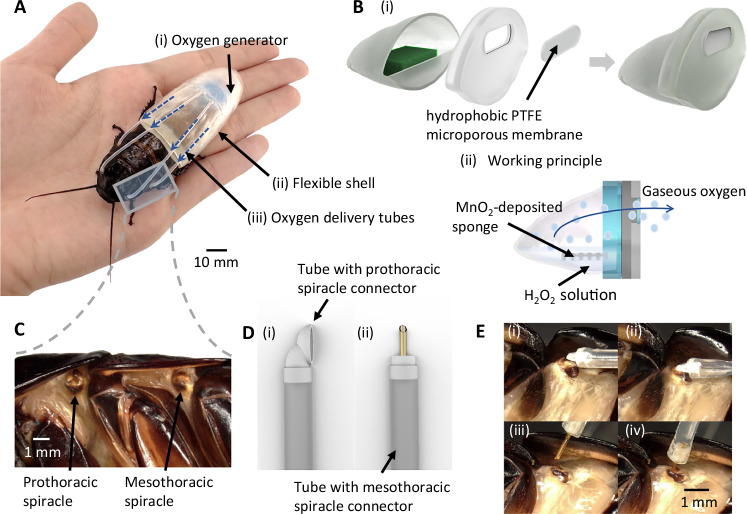
Structure and oxygen delivery mechanism of diving-suit system. **A** Diving suit integrates i) an oxygen generator, ii) a flexible waterproof shell and iii) oxygen delivery tubes. The generated oxygen is delivered through tubes to the thoracic spiracles via spiracle connectors, forming a sealed respiratory pathway. **B** Oxygen generator design. i) Exploded view of oxygen generator. It includes: a container with a MnO_2_-deposited cellulose sponge inside, a sealing lid, and a hydrophobic PTFE microporous membrane. ii) Working principle of hydrophobic PTFE microporous membrane, which allows gas to pass through while preventing liquid penetration. **C** Optical microscope image of thoracic spiracles. The left one is the prothoracic spiracle, which remains open with a lip-like structure. The right one is the mesothoracic spiracle, which stays closed with only a small hole open. **D** Customised spiracle connectors with oxygen delivery tubes. **E** Installation of the oxygen delivery tube with spiracle connector to i–ii) prothoracic spiracle, iii–iv) mesothoracic spiracle.

A chemical reactor-based oxygen generation unit (Fig. 2A, i), was implemented to eliminate the need for electronic components and maintain a compact, insect-mountable design. This oxygen generator unit is housed within a lightweight and flexible shell that attaches easily to the insect’s body (Fig. 2A, ii). Given that cockroaches breathe via thoracic spiracles^14^, oxygen delivery tubes were installed to connect the generated oxygen to the thoracic spiracles (Fig. 2A, iii). The tube tips were shaped for secure mechanical attachment to the spiracular valves, forming an integrated and wearable diving suit.

Oxygen was generated through the MnO_2_-catalysed decomposition of H_2_O_2_, which produces only water and oxygen as by-products and proceeds readily under neutral conditions^15–17^. When MnO_2_ powder was directly mixed with H_2_O_2_ solution in the confined 1.6 ml reactor caused rapid decomposition, vigorous bubbling and fluid agitation^15,18^, which destabilised the cockroach’s movement. Hence, MnO_2_ was deposited onto a highly absorbent hydrophilic cellulose sponge (1 × 1 cm). This configuration confined the reaction to solid–liquid interfaces, where oxygen was generated from numerous separated microsites (Supplementary Fig. S1), preventing gas accumulation and large-bubble coalescence. To prevent liquid agitation and ensure stable oxygen release, H_2_O_2_ was dripped onto the sponge, which serves as a carrier for the H_2_O_2_ solution and a substrate for MnO_2_ deposition. To ensure the safe operation of oxygen generators near insects, the generator structure must prevent chemical leakage and transport only oxygen to the outside shell. The oxygen generator comprises the small container that housed MnO_2_-deposited sponge and a lid that incorporated hydrophobic PTFE microporous membrane with a pore size of 0.22 µm (Fig. 2B, i). The micropores allow gas to permeate but block liquid penetration. The membrane was integrated into the lid, allowing the gaseous oxygen alone pass through and be released outside while remaining H_2_O_2_ solution, MnO_2_ powder (mostly 1–10 µm in diameter, larger than the pore size), and the generated liquid water were retained inside the generator, eliminating risk of chemical leakage (Fig. 2B, ii). Sealing stability of the oxygen generator was confirmed by subjecting the assembled unit to agitation on a vortex shaker for 10 min to simulate mechanical shocks. Afterward, the lid surface was wiped with water-sensitive test paper, and no colour change was observed. That unit was then placed with the lid and membrane facing downward over another piece of water-sensitive test paper for 3 h. No colour change was detected on the paper, confirming that no liquid leakage occurred. To further evaluate potential biological impact on long-term exposure to byproducts of H_2_O_2_ decomposition, five diving suit-wearing cyborg insects were monitored for three days following experimental exposure. All individuals survived throughout the observation period with normal behaviours. Because the decomposition of H_2_O_2_ is exothermic^19^, excessive heat could disturb the insect’s physiology. However, no noticeable temperature rise was detected at the oxygen generator when monitored with an infrared camera (Ti400, Fluke), with the temperature remaining between 23.6 and 24.0 °C throughout the reaction (Supplementary Fig. S2). This result suggested that the dispersed MnO_2_ catalytic sites on the cellulose sponge and the utilisation of small reactant amounts (2 mg MnO_2_ powder and 3% H_2_O_2_ solution) minimised the heat release, prevented localised heat accumulation and allowed the generated heat to diffuse without affecting the surrounding temperature. Future improvements in oxygen generators could focus on actively regulating oxygen generation rates. For example, integrating miniature oxygen concentration sensors and micropumps would enable quantitative delivery of H_2_O_2_ based on real-time oxygen levels within the suit, thereby achieving dynamic oxygen supply matched to the insect’s activity states and overcoming limitations of the current passive system.

Initially, dorsal mounting of the oxygen generator on the cockroach created significant water-resistance during underwater locomotion and raised the centre of gravity to approximately 1.7 cm, causing postural instability and rollover. The ventral side provided only a limited gap of 2–3 mm from the ground, insufficient to accommodate the oxygen generator there. To preserve the insect’s streamlined body profile and maintain a low centre of gravity, the generator was therefore positioned at the posterior end of the abdomen and secured by enclosing both the generator and the abdomen within the lightweight and flexible shell (Fig. 2A, i). This configuration enabled stable and smooth underwater walking without rollover.

The shell was designed as a thin-walled (1 mm), flexible enclosure that wraps around the cockroach’s abdomen, enabling fabrication with flexible resin, preserving natural movement, and accommodating individuals with slight size variations (Fig. 2A, ii). The shell geometry was modelled on the natural morphology of the cockroach’s abdomen, incorporating an oval cone shell led to the least impact on the cockroach’s movement^20^ and maintained the streamlined body form of the cockroach. The cone’s wide opening allowed the shell to slide smoothly over the tapered abdomen from the posterior end, making the installation straightforward. The anterior end of the shell was sealed to the cockroach’s first abdominal segment with a soft nitrile rubber membrane (0.16 mm thick, 1.0 cm wide). The membrane filled the narrow gap between the shell and the exoskeleton surface, forming an elastic seal that prevented water ingress and maintained a watertight interface. Its flexibility allowed the membrane both to accommodate slight variations in abdominal size among individuals and to deform with the cockroach’s body during locomotion (Supplementary Fig. S3), thereby preserving natural vertical and lateral movement.

The oxygen delivery tubes transport oxygen from the oxygen generator inside the shell to the thoracic spiracles (Fig. 2A, iii). One end of the tubes connects to the shell; the other end connects to the spiracles. The external, soft and anatomically distinct nature of the two pairs of thoracic spiracles (Fig. 2C) presents a design challenge^14^. The prothoracic spiracles remain open, forming a groove that directly exposes the spiracular valve, whereas the mesothoracic spiracles remain closed, leaving only a small hole visible. This complexity mandated the design of customised connectors to achieve a tight seal that ensures both effective oxygen delivery and waterproof sealing. The prothoracic spiracles connector has a spoon-shaped cover with an oval cap (Fig. 2D, i), designed to fully enclose the spiracular valve exposed at the prothoracic spiracle (Fig. 2E, i, ii). Conversely, the mesothoracic spiracles connector has a thin tube (ID = 0.3 mm, OD = 0.4 mm) (Fig. 2D, ii), which can be inserted into the small hole (Fig. 2E, iii, iv). Upon attachment to the spiracles, these spiracular connectors prevent water from entering the respiratory system, ensuring stable oxygen transport.

The diving suit’s waterproof performance was validated through immersion and mechanical bending tests (See Method 3.5). Waterproof integrity was confirmed after 30 min of immersion and repeated joint bending, the water-sensitive paper placed inside the suit showed only a small colour change (Supplementary Fig. S4), without the typical blue coloration that indicates water penetration. A control test performed in air produced a small color change (Supplementary Fig. S4), confirming that the observed change originated from the insect’s respiratory moisture rather than from external leakage. These results substantiate the suit’s ability to maintain a waterproof barrier under long-term immersion and mechanical deformation. Multi-directional drop tests were conducted to assess tolerance to accidental mechanical impact^21,22^. The cyborg insects with diving suits were released from heights ranging from 20 cm to 1 m in various postures, simulating real-world fall and collision scenarios. Throughout all tests, the diving suit structure remained intact without damage, the oxygen generator operated without leakage, and the cockroach remained active and capable of responding to external stimuli, demonstrating adaptability to mechanical impact. Furthermore, the suit’s performance under different water levels was evaluated by immersing the cyborg insects with a diving suit to depths ranging from 5 to 50 cm (approximately 20 times the body length). The diving suit maintained structural integrity at all test depths without deformation, and the internal test paper exhibited no blue colour change, confirming that no leakage occurred under increased water pressure. Meanwhile, the cockroach remained alive and exhibited normal locomotion across all depths.

Overall, the integration of the miniature H_2_O_2_–MnO_2_ based oxygen generator, flexible shell, and specially designed oxygen delivery tubes facilitates stable underwater respiration and locomotion while preserving natural behaviour. These outcomes confirm that the diving suit enables the cyborg insects to maintain respiration, waterproofing, and mechanical resilience underwater.

Moreover, the proposed diving suit concept could be potentially extended to other terrestrial cyborg insect platforms, such as cockroaches^23,43^, locusts^24,25^, and beetles^26,27^. These insects share similar body structures and tracheal respiratory systems^28–34^ in which oxygen enters through paired spiracles and is distributed through internal tracheal networks^35^. These similarities suggest that the strategy of combining an abdominal protective shell with oxygen delivery to the spiracles may also be applicable to terrestrial insect species. However, species-specific differences in morphology, locomotive behaviour, and payload capacity may introduce engineering challenges. For example, jumping insects such as locusts may require optimisation of suit weight and hydrodynamic design, while insects with wings folded along the abdomen may require modified shell geometries to avoid interference with wing deployment. In addition, species with limited payload capacity may necessitate further miniaturisation of the oxygen generation system and the use of ultralight materials. Therefore, adapting the diving suit to different insect species will likely require species-specific optimisation of structural design and device miniaturisation. Such adaptations could further broaden the applicability of the diving suit to diverse terrestrial insects, enabling a wider range of amphibious cyborg insect platforms.

### In-suit oxygen concentration monitoring and metabolic rate

**Fig. 3:**
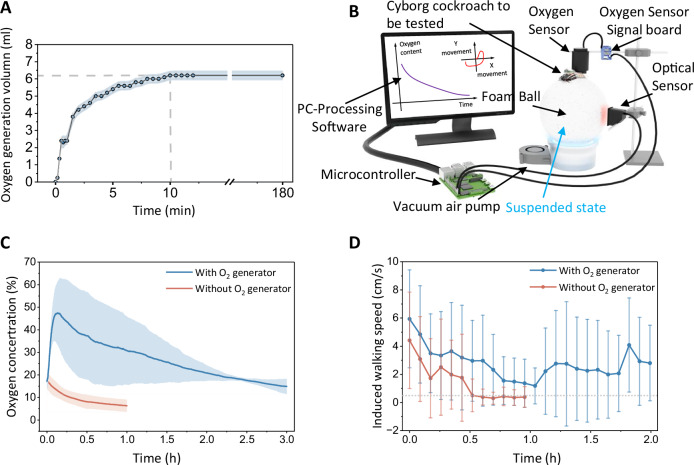
Evaluation of oxygen generation performance. **A** Oxygen generation volume (mean ± standard deviation, *n* = 3). **B** Apparatus for measuring oxygen concentration in the suits and cockroach activity. The cyborg cockroach was attached to the support system such that the animal was positioned on the centre of the Styrofoam ball **C** Oxygen level in the suits with and without oxygen generation system. Solid lines indicate the mean O_2_ level, and the shaded area indicates the standard deviation. **D** Locomotor activity of cockroach. The thick lines depict the mean value, and vertical lines indicate the standard deviation. The horizontal dotted line represents a walking speed of 0.5 cm/s.

To assess the impact of the miniaturised oxygen generator on cockroach respiration and locomotor abilities, the oxygen generation rate, oxygen consumption rate, and in-suit oxygen concentration were measured. The introduction of 1.0 ml of 3% H_2_O_2_ to the oxygen generator yielded a cumulative oxygen production of 6.2 ± 0.2 ml (Fig. 3A). To evaluate the capability of the diving suit and oxygen generator, the oxygen level in the suit was measured while the cyborg cockroach walked on a treadmill (Fig. 3B). The oxygen level inside the suits increased and reached the peak at 8.0 min after injection of 1.0 ml H_2_O_2_ (47.4 ± 14.2%, mean ± standard deviation, *N* = 6) (Fig. 3C, blue line). Thereafter, the oxygen level gradually decreased, reaching 14.8 ± 3.4% after 3 hours. Such an oxygen concentration is sufficient for insects to sustain normal physiological function^36^.

In the control test without H_2_O_2_ introduction, respiratory oxygen consumption caused the oxygen level inside the suit to continuously deplete, dropping to 6.2 ± 2.7% (mean ± standard deviation, *N* = 4) in 1 h (Fig. 3C, red line). Given that oxygen levels below 5% suppress insects activity and can lead to mortality^37,38^, operation of cyborg cockroaches without the generator is limited to less than 1 h. Reaction to electrical stimulation revealed the enhanced locomotor activity under maintained oxygen levels (Fig. 3D). During the first 5 min (i.e., first ten stimulations), cockroach with oxygen supply showed the highest response with the induced speed of 5.9 ± 3.5 cm/s. Despite the expected decline in speed due to habituation, induced forward movement was sustained above 1.4 cm/s even after 2 h (Fig. 3D, blue line). The induced speed in the group without oxygen supply fell below 0.5 cm/s after 30 min, indicating that the cockroach was deemed to stop responding^39^. The total walking duration was 28.8 ± 7.6 min with oxygen supply and 11.9 ± 7.4 min without it. These results confirmed that oxygen supply in the suits maintained prolonged locomotor activity of cyborg cockroaches. The oxygen requirement of the cyborg cockroach was also determined (See Method 3.7). At rest, they consumed 2.3 ± 0.4 ml/h (mean ± standard error, n = 12, *N* = 3), rising to 3.8 ± 0.6 ml/h (mean ± standard error, n = 12, *N* = 3) while walking. Considering oxygen production rate (Fig. 3A), the estimated survival time exceeds 1 h for continuous walking and 2–3 h while resting, even without an external oxygen supply.

### Survivability and locomotion test

The operational capability of the cyborg insects in submerged environments was evaluated following confirmation of the diving suit design and feasibility. Cyborg insects with diving suit sustained activity and responsiveness to external electrical stimuli for 2–3 h underwater (Supplementary Movie S1). In contrast, a control cockroach without the diving suit suffocated within 2 min (Supplementary Movie S1). These findings establish that the diving suit, by sealing out water and supplying oxygen, extended the cockroach’s underwater survival time from a few minutes to several hours. Furthermore, cyborg insects wearing a diving suit exhibited user-induced entry and exit from water, demonstrating prolonged controllability and maintained physiological activity across the land–water interface (Supplementary Movie S2).

**Fig. 4:**
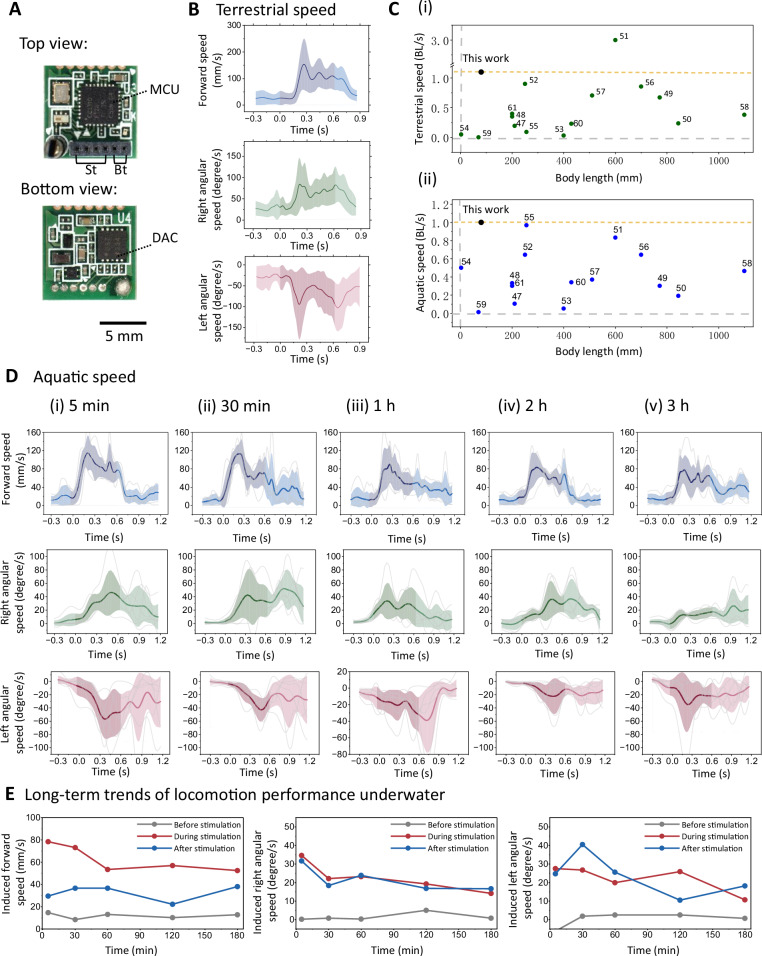
Locomotion performance of cyborg cockroaches on amphibious environment. **A** Top and bottom side of the backpack. MCU: microcontroller unit, DAC: digital-to-analog converter, St: stimulation channel, Bt: battery terminal. **B** Terrestrial speed (mean ± standard deviation, *n* = 40 (forward speed), *n* = 25 (angular speed), *N*  =  3). **C** Locomotion performance comparison of amphibious cyborg insects with other amphibious robots. BL/s is Body length/s. Numbers correspond to the cited works in the references section. i) Terrestrial speed comparison. ii) Aquatic speed comparison. **D** Aquatic speed at multiple time points after immersion (mean ± standard deviation, *n* = 6, *N* = 3). **E** Long-term trends of locomotion performance underwater (mean).

Locomotion of the cyborg insects was then evaluated on land and underwater. On land, the suit-wearing cyborg insects achieved an average forward speed of 87.5 mm/s, with left-turn angular speed of 58.6°/s and right-turn angular speed of 58.1°/s (Fig. 4B, i–iii). Underwater, the average forward speed was reduced to 78.4 mm/s, and average left-turn and right-turn angular speed declined to 35.2°/s and 28.2°/s (Fig. 4D, i). The reduction in both forward and turning speeds underwater (a 10.4% decrease for forward speed and 39.9% and 51.4% declines for left- and right-turn speeds, respectively) is primarily attributable to increased hydrodynamic resistance. This disparity, where angular speed loss is more pronounced than forward speed loss, stems from geometric and hydrodynamic factors. The elongated, elliptical body of the suit-wearing cockroach increases the frontal area exposed during turning, thereby enhancing fluid resistance^40,41^. In addition, vortex shedding and unsteady flows around the body generate extra vortex-induced drag^40,42^, resulting in greater speed loss during turning underwater.

Long-term underwater performance was assessed by monitoring locomotion activity underwater for 3 h (Fig. 4D, i–v). A gradual reduction in both forwards and angular speeds was observed, primarily attributed to physiological fatigue and stimulus habituation^39,43,44^. The forward speed diminished from 78.4 mm/s at 5 min to 52.3 mm/s after 3 h. The right-turning angular speed decreased from 27.5°/s to 10.7°/s, while the left-turning angular speed dropped from 34.6°/s to 14.1°/s. A previous study demonstrated that cyborg cockroaches achieved point-to-point terrestrial navigation at average forward and angular speeds of 29.0 mm/s and 6.95°/s^39^. In comparison, the suit-wearing cyborg insects maintained higher locomotion speeds underwater, indicating that the diving suit also enables path control in aquatic conditions.

Analysis of average speeds before, during, and after stimulation revealed further differences in aquatic locomotion (Fig. 4E). Unlike the rapid speed decay observed in air post-simulation, the underwater average post-stimulus speed (Fig. 4E, blue line), particularly during manoeuvres, occasionally surpassed the average speed during stimulation (Fig. 4E, red line). These delayed acceleration and deceleration responses result from the added-mass effect of surrounding water^45,46^. When cockroaches accelerate from rest, the surrounding fluid exerts an opposing added mass force that resists their motion. Conversely, during deceleration, an added mass force continues to exert a forward thrust, thereby prolonging the slowing-down process. Concurrently, the experiments revealed that cockroaches’ tarsal claws remained anchored to the substrate underwater, contrasting with relaxed leg positions in air. This sustained gripping behavior counteracted upward buoyancy and rotational instability caused by hydrodynamic disturbances. Consequently, cockroaches should continuously exert vigorous claw grips to maintain equilibrium. This sustained gripping behaviour may also contribute to the slower motor responses observed underwater.

The amphibious moving speed of suit-wearing cyborg insects was compared with that of reported amphibious robots by normalising speed to body length per second (BL/s)^47–61^. On land, amphibious cyborg insects achieved 1.1 BL/s, surpassing most amphibious robots (typically below 1.0 BL/s) (Fig. 4C, i). Underwater condition, our system achieved a speed of 1.0 BL/s, again outperforming most existing designs (typically below 0.8 BL/s) (Fig. 4C, ii). When compared to other centimetre-scale amphibious robots with reported angular speeds^60,62,63^, the cyborg cockroach with the diving suit exhibited a several-fold higher angular speed on both land and underwater.

Overall, the diving suit enables cyborg insects to operate in both terrestrial and aquatic environments, extending their operational scope to amphibious conditions while preserving locomotion capability.

### Demonstrations of survival, adaptation

**Fig. 5:**
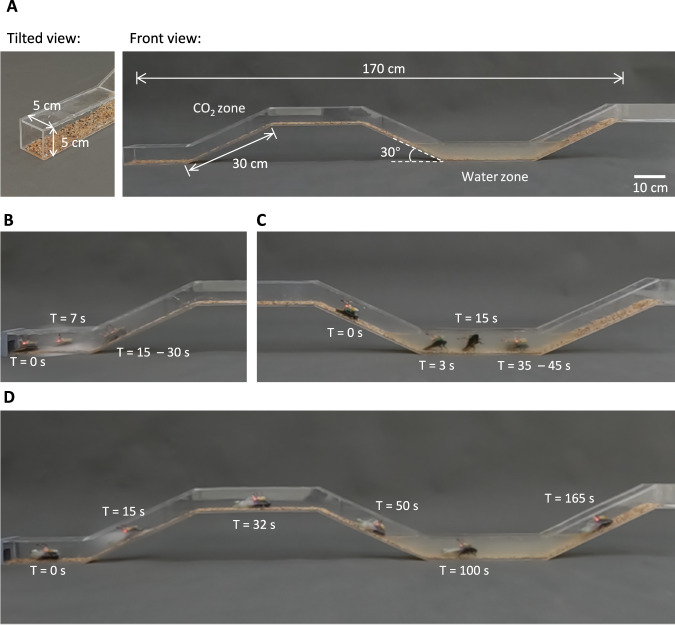
Comparison of locomotion performance of cyborg cockroaches with and without diving suit in simulated hazard environments. **A** Schematic of the tunnel setup with a length of 1.7 m and a cross-sectional size of 5 cm by 5 cm. The tunnel includes two regions: a CO_2_ zone and a water zone. **B** Locomotion sequence of cyborg cockroach without suit in CO_2_ zone. The cockroach was anaesthetized after CO_2_ exposure (we saved it by moving it to fresh air, and it recovered fully within a few minutes). **C** Locomotion sequence of cyborg cockroach in underwater zone. The cockroach became asphyxiated 45 s after entering the water (we saved it by drying and moving it to fresh air, it fully recovered within a few minutes). **D** Locomotion sequence of cyborg cockroach in tunnel with CO_2_ zone and underwater zone in Trail 1.

Operational validation required testing the diving suit’s capability to traverse simulated hazardous environments and underwater narrow crevices. To examine whether the diving suit allowed cyborg cockroaches to operate under combined hazardous conditions, they were tested in a 1.7-m-long tunnel (5 × 5 cm cross-section) composed of a CO_2_-filled section followed by a water-filled section (Fig. 5A), representing typical environmental threats—anaesthetising gases^64–66^ and full submersion. The cyborg insect without the suit exhibited disoriented movement, erratic leg movements, and loss of responsiveness to external stimuli within seconds after CO_2_ exposure (Fig. 5B, Supplementary Movie S3). Because the cockroach became immobile in the CO_2_ zone, another cyborg insect was introduced at the entrance of the water zone to evaluate its reaction to submersion. Under electrical stimulation, the cyborg insect was guided to walk into the water, where its leg movements gradually ceased, and complete immobility occurred within 45 s, suggesting asphyxia (Fig. 5C, Supplementary Movie S3). In contrast, the cyborg insect equipped with the diving suit maintained a regular gait and successfully traversed the entire tunnel (Fig. 5D, Supplementary Movie S3). Three independent trials were conducted under identical CO_2_–water tunnel conditions, and all cyborg insects wearing the diving suit successfully traversed both hazardous zones (3/3 successful trials). Locomotor activity was preserved throughout both sections, demonstrating that the diving suit enables sustained respiration and movement under combined CO_2_ exposure and water submersion by preventing gas and water ingress while supplying oxygen independently.

**Fig. 6:**
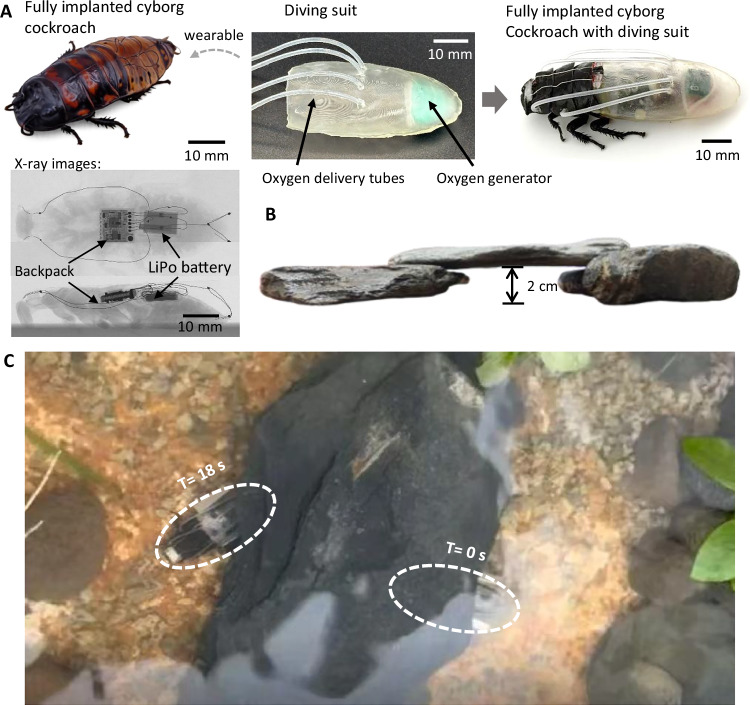
Outdoor underwater narrow crevice traversal. **A** cyborg insects under fully implanted configuration with diving suit. The backpack and battery are surgically implanted into the cockroach. The top and side views of X-ray images (bottom) confirm the internal implantation of these components. The diving suit integrates an oxygen generator and oxygen delivery tubes, providing a waterproof shell that maintains respiration and protection underwater. **B** Schematic of the constructed crevice with a height of 2 cm. **C** Locomotion sequence of a fully implanted cyborg insect with a diving suit navigating through the narrow crevice. The cyborg insect enters from the right side and exits from the left. The total length of the crevice is approximately 10 cm.

Evaluation of the cyborg insect’s capacity for manoeuvring through confined underwater spaces, necessitated testing in a narrow crevice. In submerged underwater spaces with gaps as small as 2 cm, the dorsal backpack module (Figs. 1B, 4A) protrudes from the thorax and prevents passage. The insect lacks sensory feedback to detect such obstacles and continues to push forward, eventually becoming stuck because the backpack is an external attachment. To eliminate this protrusion and enable smooth passage through confined areas, the backpack and battery were implanted inside the body, forming a fully implanted cyborg configuration (Fig. 6A)^49^. Utilising this configuration, cyborg insects with the diving suit could traverse a 2-cm-high crevice (Fig. 6B, C), which would be difficult for regular cyborg insects with thorax-mounted backpack (2.5 ± 0.3 cm) and for most aquatic robots. The fully implanted configuration also altered the internal mass distribution, relocating the backpack and battery closer to the body’s central axis and thereby lowering the centre of gravity, which improved stability and reduced the likelihood of overturning during underwater locomotion. This internal configuration also prevents the snagging or detaching of external parts when navigating narrow gaps or uneven underwater terrain. Overall, these results demonstrate that the diving suit facilitates the maintenance of respiration and movement in sequential CO_2_–water environments by preventing gas and water ingress, while providing an independent oxygen supply.

## Methods

### Materials

Rigid, flexible, and PMMA-type 3D printing resins (JAMGHE Art Engineering resin, F39T, and PMMA-like resin) were obtained from Shenzhen Yongchanghe Technology Co., Ltd., Dongguan Godsaid Technology Co., Ltd., and Shenzhen ESUN Industrial Co., Ltd. (China), respectively. Medical H_2_O_2_ 3% solution was purchased from ICM Pharma Pte Ltd (Singapore). MnO_2_ powder (99.95% metal basis) was from Macklin (China). All the chemicals were used as received. Cellulose sponge was obtained from Ningbo Denlux-Shijia Home Products Manufacturing Corp, China. Hydrophobic PTFE microporous membrane (pore size = 0.22 µm) was from Shandong Jukai Environmental Protection Technology Co., Ltd. Water-sensitive test paper was from Linyi Yinnuoweixin Laboratory Equipment Co., Ltd.

### Animals

*G.portentosa* (adult, average weight: 5.5 ± 1.0 g, average length: 6.0 ± 0.5 cm) were used in this study. The insects were reared in a mouse housing unit (NexGen Mouse 500, Allentown Inc.) equipped with a clean-air circulation system. Each 19 × 13 × 38 cm plastic container housed no more than five cockroaches. They were fed fresh carrot slices once per week, and the containers were cleaned weekly to maintain hygiene. The rearing room was maintained at a constant temperature of 25 °C and 60% relative humidity.

### Miniature wireless stimulator (Backpack)

The compact backpack is designed for ease of attachment and minimal weight (Fig. 4A). The size of the backpack is 10 × 10 mm. A microcontroller CC1310F128 serves as the main processing unit of the backpack, with a footprint of only 4 × 4 mm and ultra-low-power wireless communication capability (Sub 1-GHz with a current of 13.4 mA at 10-dBm transmit power and 5.5 mA at receive). The stimuli signal is generated by a digital-to-analogue converter (DAC) integrated circuit (IC), AD5624, with a reference voltage of 5 V and a size of 3 × 3 mm^67^. A Madagascar hissing cockroach has a maximum payload capacity of approximately 15 g^68^. The diving suit (5.5 ± 0.3 g) together with the waterproof-treated backpack (0.7 g) yielded a combined payload that remained below the established capacity threshold. The remaining weight allowance of approximately 8.8 g enables the future addition of more power sources or sensing components.

### Preparation of diving suit-wearing cyborg cockroaches

The induction of movement in the cyborg cockroach was based on an established procedure^69^. To facilitate electrode implantation, cockroaches were anesthetised with carbon dioxide for approximately 30 s in a sealed container. Once anesthetised, the distal tips of both antennae and cerci were trimmed to create small incisions. Prior to implantation, the insulation of Teflon-coated silver wires (786000, AD Instruments) was carefully removed with a lighter to expose the conductive core. The prepared wires were inserted to a depth of 5 mm, secured with beeswax, and the openings were sealed to prevent infection. For implantation in the third abdominal segment, small holes were made with an insect pin at the designated sites, and the silver wires were inserted perpendicularly into the exoskeleton to a depth of 5 mm. After implantation, the openings were sealed again with beeswax. The free ends of the implanted wires were connected to the wireless stimulator, completing the electrode interface for locomotion control. The underwater diving suit consisted of three components: an oxygen-generation tank, a flexible shell, and four silicone oxygen-supply tubes. The assembly process is illustrated in Supplementary Fig. S5. The oxygen generator tank was 3D-printed with PMMA-type resin, with MnO_2_–deposited sponge integrated inside. The lid vent was covered by a hydrophobic PTFE microporous membrane. The flexible shell was customised to match the morphology of the cockroach abdomen and sealed to the first abdominal segment with a nitrile rubber membrane using adhesive. After experiments, the shell can be removed and the membrane can be gently polished off to minimise any restrictions on the cockroach’s normal behaviour and daily activities. Four small dorsal openings were reserved for attaching the oxygen tubes, which were connected to the thoracic spiracles. Before attachment, the spiracular surfaces were cleaned and lightly roughened, followed by application of a small amount of adhesive to improve bonding.

Two 3D-printed spiracle connectors were fabricated to accommodate anatomical differences. These connectors were secured with adhesive, ensuring stable gas transfer and complete waterproofing. To stabilise the tubing, small anchoring holes were drilled into the thoracic exoskeleton, preventing interference from leg motion during locomotion. After assembly, visual inspection and short-duration immersion tests were conducted to verify sealing integrity. Units that failed inspection were repaired and reinstalled. Finally, 1.0 mL of 3% hydrogen peroxide was introduced into the MnO_2_-deposited sponge through the shell’s injection hole to initiate catalytic oxygen generation, after which the shell was sealed with UV adhesive, forming an enclosed oxygen-supply cavity that allowed the cyborg cockroach to survive and move underwater for extended periods.

### Evaluation of waterproof under immersion and bending

To assess the long-term waterproof performance of the diving suit under realistic operational conditions, a cyborg cockroach wearing the suit was completely immersed in water for 30 min. During immersion, the thoracoabdominal joint, where the flexible shell attaches to the body, was continuously flexed to simulate the mechanical deformation occurring during natural locomotion. To detect any possible water ingress, water-sensitive test paper (1 × 2 cm) that turns blue upon contact with moisture was placed inside the suit near the thoracoabdominal junction. After immersion, the test paper was removed and examined for colour change. To distinguish genuine leakage from humidity-induced discoloration, a control test in air was conducted for 30 minutes under identical conditions. This procedure enabled clear differentiation between colour changes caused by external water intrusion and those resulting from the insect’s respiratory moisture.

### Preparation and monitoring of the oxygen generation system

Oxygen generation for sustaining cockroach survival in a submerged yet gas-filled enclosed environment was achieved via the catalytic decomposition of H_2_O_2_ by MnO_2_. To enable controlled and sustained oxygen release, MnO_2_ was immobilised onto a cellulose sponge substrate, forming an MnO_2_-deposited sponge. The MnO_2_ powders were confined within the pores of the cellulose sponge, preventing them from dispersing freely. The fabrication process involved dispersing MnO_2_ powder in 15 MΩ·cm deionised water (MilliQ IX7005, USA), followed by ultrasonication for 15 min to reduce particle size and enhance deposition uniformity. The resulting MnO_2_ dispersion was then drop-cast onto a cellulose sponge pad (10 × 10 mm) and dried in an air oven at 45 °C for at least 3 h before use. Upon the addition of 1.0 ml 3% H_2_O_2_ solution, the sponge structure absorbed the liquid, triggering the catalytic decomposition reaction and generating oxygen.

The efficiency and stability of the oxygen generation system were evaluated by continuously monitoring the in-suit oxygen concentration while a cyborg cockroach walked on a treadmill (Fig. 3B). An oxygen sensor (AO2 CiTicel, City Technology) was attached to the suit using a 3D-printed connector and sealed with hot glue. The sensor was connected to a microcomputer (Arduino DUE, Arduino) via a signal board (SEN0496, DFRobot). An optical motion sensor, positioned near the treadmill (Styrofoam ball, 12 cm in diameter, 25.8 g in weight), measured the cockroach’s movement and was linked to the same microcomputer. The sampling rate for both oxygen and optical sensors was set to 5 Hz. Electrical stimulation was applied every 30 s via platinum electrodes implanted into the cerci, delivering 2.5 V pulses controlled by the stimulator module. A custom-written program was used to operate the controller and record oxygen data. The oxygen sensor was calibrated before each experiment using single-point calibration in ambient air (20.9% O_2_).

### Measurement of oxygen generation rate and oxygen consumption rate

The oxygen generation rate was determined using a water displacement gas collection apparatus (Supplementary Fig. S6). Oxygen produced by the reaction between MnO_2_ and H_2_O_2_ displaced water within the gas measuring tube, with the gas volume recorded over time. The oxygen consumption rate was measured using an open-flow respirometry system (Q-box RL1LP, Qubit Systems). The cyborg cockroach equipped with the diving suit was connected to the system through two silicone tubes (inlet and outlet, each 30 cm in length, with an outer diameter of 3 mm and an inner diameter of 2 mm). The cockroach was placed in a plastic chamber lined with small plastic plates to provide a stable walking surface. During the experiment, the plates were gently adjusted to prevent the silicone tubes from impeding the cockroach’s natural movement. To induce locomotion, electrical pulses of 1.5 V were applied to the cerci every 15 s via the control system.

### Underwater locomotion performance test

At room temperature, locomotion experiments were performed in a 50 × 50 cm water tank. Forward and turning movements were induced by applying electrical pulses (3–4 V, 0.6 s duration) to cyborg cockroaches with the diving suit via backpack. Control commands were transmitted from a computer through a central control unit. Trajectories of movement were recorded using an overhead HD webcam (C920 Pro, Logitech) and analysed with DLTdv8a software⁵⁸. From these trajectories, forward speed and angular speeds for left and right turns were calculated. To compensate for buoyant forces during underwater operation, a 5 g weight was attached to cyborg cockroach with the diving suit.

### Demonstrations of environmental adaptability and functional extension

#### Mixed-hazard tunnel traversal

A tunnel was constructed to simulate extreme mixed environments. The tunnel was *1.7 *m long with an internal cross-section of 5 × 5 cm, built from transparent acrylic panels to allow direct observation and video recording. Its interior alternated between horizontal and inclined sections (30° slope) to create a complex terrain (Fig. 5A). The tunnel consisted of two continuous zones: one filled with carbon dioxide (CO_2_) and the other with water. Cyborg cockroaches were placed at the entrance and guided by electrical stimulation to pass through both sections. Locomotion was recorded by three action cameras (GoPro 10) positioned on the left, centre, and right sides of the tunnel.

#### Narrow-crevice traversal in outdoor pond

An outdoor pond (50 × 50 cm, depth 10 cm) was prepared to evaluate traversal ability in confined aquatic environments. Fully implanted cyborg cockroaches, with controller and battery surgically embedded within the cockroach to eliminate external protrusions, were employed^49^. The insects were tasked with moving underwater through a stone passage only 2 cm in height, simulating a constricted gap (Fig. 6B). Their underwater motion was continuously recorded using a HD webcam (C920 Pro, Logitech).

## Supplementary information

Supplementary Information

Description of Additional Supplementary Files

Supplementary Movie 1

Supplementary Movie 2

Supplementary Movie 3

Supplementary Movie 4

Transparent Peer Review file

## Data Availability

All data supporting the findings of this study are available within the article and its supplementary files. Source data are provided with this paper.
