# Supplementary material for: Underwater Suit-Wearing Cyborg Insect Capable of Hours-Long Diving and Terra-Aqua Travel
Source: Nat Commun. 2026 Jun 29;17:5398. doi: 10.1038/s41467-026-74235-1 (PMC13315876; doi:10.1038/s41467-026-74235-1)
Supplement: Supplementary file 1 — Supplementary Information [file 41467_2026_74235_MOESM1_ESM.pdf]

Supplementary Materials for

Title:

Underwater Suit-Wearing Cyborg Insect Capable of Hours-Long Diving and Terra-Aqua Travel

Authors:

Zifu FAN[1], Kazuki KAI[1], Kewei SONG[1], Duc Long LE[1], Thu Ha TRAN[1], Mingyu HAO[2], Wei Yang WAN[1], Shinjiro UMEZU[3], Hirotaka SATO[1, *]

Affiliations:

[1]School of Mechanical and Aerospace Engineering, Nanyang Technological University; Singapore 637460, Singapore.

[2] School of Electrical and Electronic Engineering, Nanyang Technological University; Singapore 639798, Singapore.

[3]School of Creative Science and Engineering, Waseda University; Tokyo 169-8555, Japan.

*Corresponding author. E-mail: hirosato@ntu.edu.sg

The PDF file includes:

Fig. S1. Characterization of MnO_2-deposited cellulose sponge.

Fig. S2. Thermal monitoring of the oxygen generator during MnO_2 catalytic decomposition of H_2O_2.

Fig. S3. Compatibility of the diving suit with abdominal movements.

Fig. S4. Waterproof performance of the diving suit evaluated with water-sensitive test paper.

Fig. S5. Assembly Process of Cyborg Insects Equipped with a Diving Suit.

Fig. S6. Apparatus for measuring the volume of oxygen generated.

Other Supplementary Materials for this manuscript include the following:

Supplementary Movies S1-4.

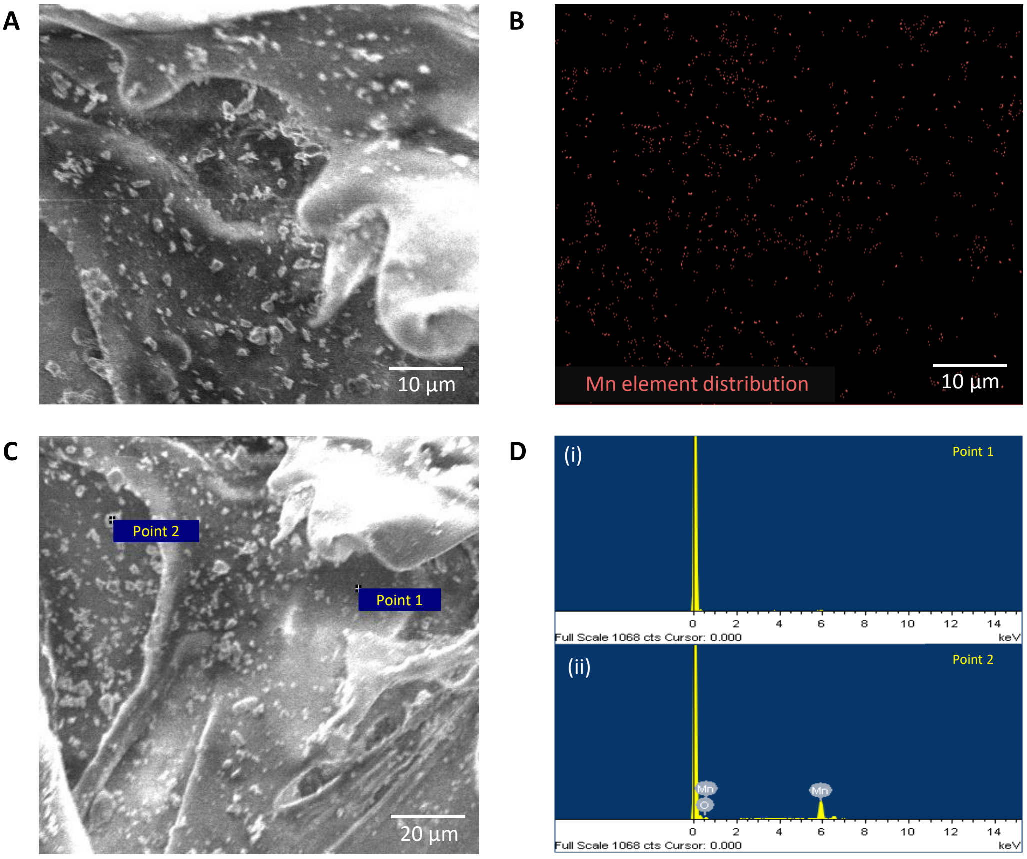

Fig. S1. Characterization of MnO_2-deposited cellulose sponge. (A) SEM image of the MnO_2-deposited sponge prepared by dispersing 2 mg of MnO_2 powder onto a highly absorbent hydrophilic cellulose sponge. (B) EDS elemental mapping of Mn element corresponding to the SEM image in (A), confirming the MnO_2 powders were distributed across the sponge fibres. (C) SEM image, showing the selected points for spectrum analysis. Point 1 represents the blank area, while Point 2 represents to the MnO_2 powder. (D) i) EDS spectrum of point 1, showing no Mn peaks. ii) EDS spectrum of point 2, identifying prominent Mn peaks, indicating that the powder observed in the SEM image is MnO_2.

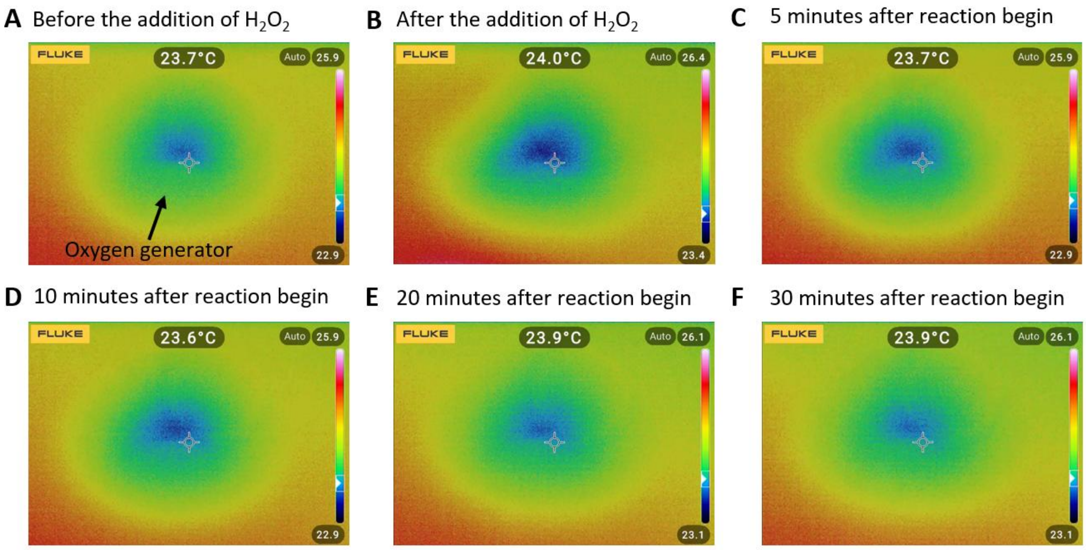

Fig. S2. Thermal monitoring of the oxygen generator during MnO_2 catalytic decomposition of H_2O_2. Infrared thermal images recorded using a Fluke Ti400 thermal imager showing temperature distribution around the oxygen generator at different time. (A) Before the addition of H_2O_2, only the MnO_2-deposited cellulose sponge was inside the oxygen generator. (B) Immediately after adding the H_2O_2 solution (3 %, 1 ml). (C–F) Temperature profiles at 5, 10, 20, and 30 min after the reaction began, respectively. The temperature remained between 23.6 °C and 24.0 °C throughout the process.

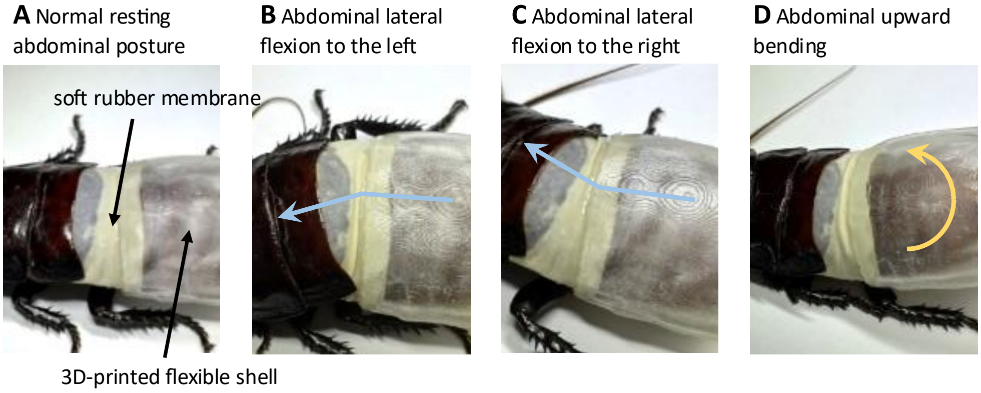

Fig. S3. Compatibility of the diving suit with abdominal movements. (A) Normal resting posture. (B–C) Lateral flexion to the left and right. (D) Upward bending of the abdomen.

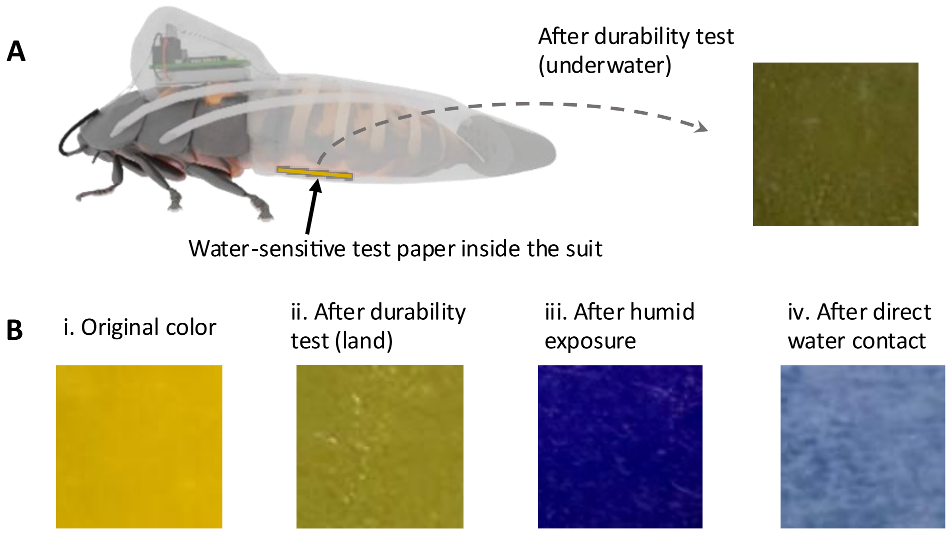

Fig. S4. Waterproof performance of the diving suit evaluated with water-sensitive test paper. (A) Placement of water-sensitive paper inside the suit during immersion testing. After underwater durability testing, the paper maintained its original yellowish colour, indicating no water infiltration. (B) Reference colour changes of the test paper under different conditions. i) original colour test paper. ii) test paper placed inside the suit on land. iii) test paper exposed to a humid environment. iv) test paper directly contacted with water.

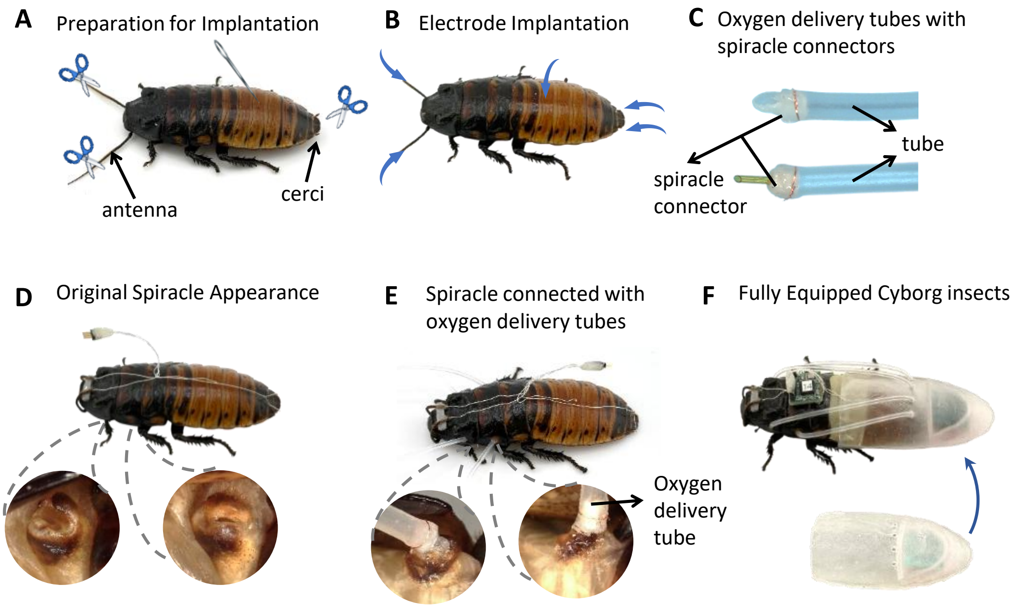

Fig. S5. Assembly process of cyborg insects equipped with a diving suit. (A) Preparation for electrode implantation. The antennae and cerci were trimmed at the locations indicated in the figure, and a hole was drilled in the cockroach's back. (B) Electrode implantation positions and directions. Silver electrodes were inserted 5 mm deep at the locations indicated by the arrows. (C) Oxygen delivery tubes with spiracle connectors. (D) Optical microscope images of the thoracic spiracles. (E) Connection of the thoracic spiracles and oxygen delivery tubes, secured with strong adhesive. (F) Fully assembled cyborg insect with diving suit. The MnO_2-deposited sponge was placed inside the oxygen generator, which is then inserted into the flexible shell. The shell is securely attached to the cockroach’s abdomen through a soft nitrile rubber membrane.

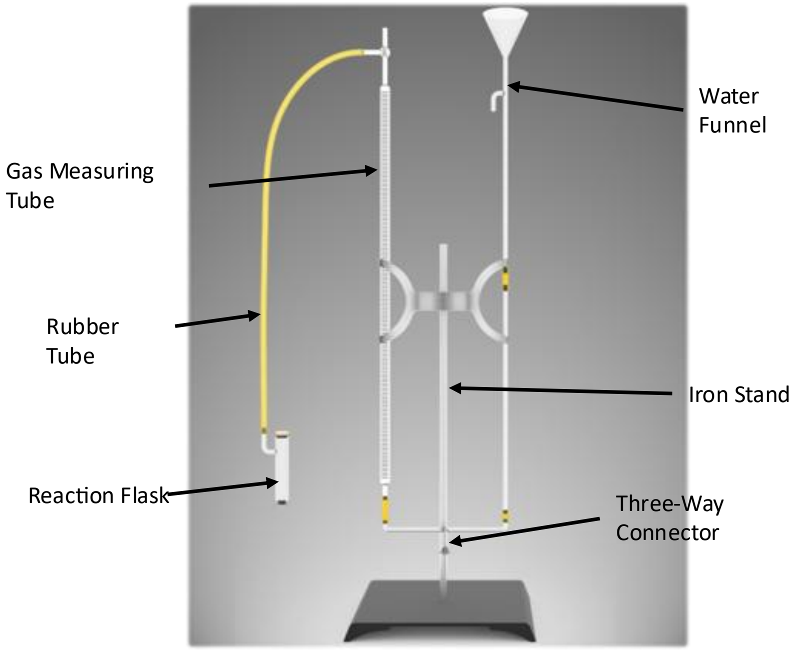

Fig. S6. Apparatus for measuring the volume of oxygen generated. The apparatus includes a water funnel, gas measuring tube, three-way connector, rubber tubes, and reaction flask.
