# Supplementary material for: Underwater Suit-Wearing Cyborg Insect Capable of Hours-Long Diving and Terra-Aqua Travel
Source: Nat Commun. 2026 Jun 29;17:5398. doi: 10.1038/s41467-026-74235-1 (PMC13315876; doi:10.1038/s41467-026-74235-1)
Supplement: Supplementary file 2 — Description of Additional Supplementary Files [file 41467_2026_74235_MOESM2_ESM.pdf]

# <u>Description of Additional Supplementary Files</u>

**Supplementary Movie 1:** Comparison of underwater survival of cyborg cockroaches with and without the diving suit.

**Supplementary Movie 2:** Seamless landunderwater-land travel.

**Supplementary Movie 3:** Survival and locomotion of cyborg cockroaches with and without diving suit in a CO2-water tunnel.

**Supplementary Movie 4:** Underwater narrow gap crossing.
