# Supplementary material for: Underwater Suit-Wearing Cyborg Insect Capable of Hours-Long Diving and Terra-Aqua Travel
Source: Nat Commun. 2026 Jun 29;17:5398. doi: 10.1038/s41467-026-74235-1 (PMC13315876; doi:10.1038/s41467-026-74235-1)
Supplement: Supplementary file 7 — Transparent Peer Review file [file 41467_2026_74235_MOESM7_ESM.pdf]

nature portfolio

# Peer Review File

# Underwater Suit-Wearing Cyborg Insect Capable of Hours-Long Diving and Terra-Aqua Travel

Corresponding Author: Professor Hirotaka Sato

**This file contains all reviewer reports in order by version, followed by all author rebuttals in order by version.**

Version 0:

Reviewer comments:

Reviewer #1

(Remarks to the Author)
The paper “Underwater Suit-Wearing Cyborg Insect Capable of Hours-Long Diving and Terra-Aqua Travel” by Fan et al. presents a cyborg insect system where Madagascar hissing cockroaches are equipped with a wearable underwater suit that enables survival underwater for several hours. The suit features a 3D-printed shell and an oxygen generation system using $MnO_2$ and $H_2O_2$, aiming to support insect-based exploration in hazardous environments.
The introduction reads more like a review and lacks a focused scientific question or hypothesis. For a high-impact journal like Nature Communications, the study must clearly state the novelty and significance in a hypothesis-driven context. Right now, it feels exploratory without a clear experimental narrative.
The manuscript is excessively descriptive—especially in sections detailing insect anatomy and the oxygen supply mechanism—without enough quantitative analysis, control experiments, or statistical treatment of data. No empirical data is provided on oxygen levels maintained inside the shell. There’s no mention of how long the cockroaches survived in water with and without the suit in controlled tests. No comparison to existing solutions or systems is presented quantitatively.
There is a major gap in demonstrating real-world utility and functional testing of the cyborg system. Has the suit been tested in simulated hazardous environments (e.g., smoky, flooded tunnels)? What metrics were used to assess system performance, endurance, and behavior under stress? Without such validations, the claim of applicability in extreme environments remains speculative.
Using $MnO_2$ and $H_2O_2$ in proximity to living organisms raises significant concerns regarding toxicity, heat generation, and potential harm to the insect. No discussion is provided on the possible biological impact of long-term exposure to reaction byproducts. The environmental safety of deploying such cyborgs with chemical tanks in natural disaster zones is unaddressed.
The manuscript suffers from repetitive phrasing, unclear transitions, and poor figure referencing (e.g., “Fig. S1” appears without prior context). Additionally, the structure lacks coherence in moving from background to methodology to result interpretation. Critical technical details are buried within long paragraphs without proper emphasis or figure cross-referencing.
Several claims are overreaching or unsubstantiated.
"First time a cyborg insect can survive underwater for several hours" – needs clear prior-art review and references to justify this claim.
"Lays the groundwork for exploration in extreme environments" – without field testing, this is not credible at this stage. High-impact journals require not just novelty but rigor and restraint in claims.
English needs a deep revision.

Reviewer #2

(Remarks to the Author)
This study presents an innovative biohybrid system combining a Madagascar hissing cockroach with a wearable underwater suit, enabling prolonged amphibious operation. The integration of an oxygen supply system ($MnO_2$-$H_2O_2$ reaction) and a 3D-printed flexible shell demonstrates potential for search-and-rescue and environmental monitoring applications. While the work is groundbreaking, major revisions are required to address experimental gaps. Below are detailed critiques and recommendations.
Major points:

1. Oxygen Supply System:
Insect locomotion as well as energy consumption tends to be stronger in the electrically stimulated state than in the unstimulated state. Therefore, the $MnO_2$-$H_2O_2$ reaction rates should be correlated with the cockroach's metabolic demand across different activity states (with or without electrical stimulation). Plot oxygen consumption vs. generation rates dynamically.
2. wearable underwater suit:
(1) Although the morphology of the underwater suit was designed with reference to the real form of the cockroach abdomen, did the design of the dimensions take into account the effects on the cockroach's locomotor behavior and the deformation of the abdomen?
(2) The risk of catalyst failure or liquid leakage during the operation of cockroaches underwater has not been evaluated, and durability experiments on the sealing of waterproof clothing underwater need to be increased.
3. Locomotion control:
Although the authors mentioned the motion attenuation of cockroaches under water. It is suggested to further explore how to improve the speed and stability of cockroach's movement in underwater environments and how to extend its task execution time.

Additional Weaknesses:
1. The manuscript lacks quantitative comparisons with existing amphibious robots (e.g., energy efficiency, speed, payload capacity).
2. The introduction of experimental methods in Result and Discussion section is redundant and streamlining is recommended.

Reviewer #3

(Remarks to the Author)
** General Assessment **

This manuscript presents an innovative system enabling amphibious capabilities in cyborg insects using a wearable underwater suit that integrates an oxygen generation system. The authors demonstrate that Madagascar hissing cockroaches can survive and manoeuvre underwater while carrying control and sensing modules. The concept is novel, and the experiments are generally well described. However, several claims need stronger experimental support, and further clarification is needed in multiple areas.

** Major Comments **

1) The authors state that the cyborg insects "can operate effectively on land while also surviving underwater for several hours." However, the main data presented focuses on underwater operation. Please clarify whether there is experimental evidence that the insects with the suit and control systems can still navigate on land. If such data exists, please include it; if not, the claim should be rephrased.

2) The title references "hours-long diving." The data in Figure 2c and 2d show oxygen concentration and movement lasting up to 3 hours. Please confirm whether these data represent continuous, active underwater navigation and clarify what tasks were performed during that period. This would better support the claim.

3) A comparison with the state of the art is missing. Please contextualize the performance of your system in terms of speed and maneuverability --- both in air and water --- relative to other cyborg insect systems or miniaturized amphibious robots.

4) The manuscript does not mention how reliably the system works across trials. What was the success rate for underwater operation? Were there any observed failure modes such as water leakage or system malfunctions? Please comment on robustness.

5) Lines 122–136 discuss the MnO2-H2O2 oxygen generation system. While the duration of oxygen generation is compared to metabolic demands, the actual oxygen volumes generated and consumed are not clearly matched. Please include these data to support the claim that oxygen generation was sufficient.

6) The Results section contains a large amount of technical detail that may be better suited for the Methods section. For example, the content in lines 236–278 describing the modeling and printing steps could be condensed or moved for clarity.

** Minor Comments **

- Figures 1 and 4 would benefit from the inclusion of scale bars.

- The supplementary movie should be clearly referenced in the text.

- Line 59: Please clarify the "disadvantage compared to insects when size matters."

- Line 62: Dragonflies are generally classified as aerial insects. Please revise.

- Line 134: Using km/h for cockroach speed seems unusual; cm/s would be more appropriate.

- Line 137: The phrase “traditional oxygen generation systems” would benefit from a brief explanation of what those systems are, for comparison.

- Line 187: Clarify how the “first 5 minutes” of walking velocity was computed --- was it a time average, or sampled at a specific time?

- Line 215: Please elaborate on how structural efficiency and adaptability were evaluated during modeling.

- Line 328: The comparison to previously measured air-based movement is unclear. Why was this data not measured as part of the current study? Please clarify or provide the relevant new data.

Reviewer #4

(Remarks to the Author)
I co-reviewed this manuscript with one of the reviewers who provided the listed reports. This is part of the Nature Communications initiative to facilitate training in peer review and to provide appropriate recognition for Early Career Researchers who co-review manuscripts.

Version 1:

Reviewer comments:

Reviewer #2

(Remarks to the Author)
The authors have made substantial efforts to address the concerns raised in the initial review, with significant additions of experimental data, clarifications, and textual improvements. The revisions have greatly enhanced the rigor and clarity of the work. The authors have added critical quantitative data, such as oxygen level monitoring, survival comparisons, and performance benchmarks against existing amphibious robots. The hypothesis-driven restructuring of the introduction and the professional language editing also improve the manuscript’s impact. While minor issues remain, the study now demonstrates novelty, methodological robustness, and potential applications in extreme environments, meeting the high standards of Nature Communications. While the revisions are commendable, the following points should be addressed in a minor revision:
(1) The authors note that the system lacks real-time oxygen regulation to match metabolic demands during activity states. Although insects tolerate oxygen fluctuations, the manuscript should discuss this limitation and propose feedback-controlled systems as future work.
(2) While reagent confinement minimizes risks, data on long-term exposure to byproducts are indirect. Adding a sentence on post-experiment insect recovery would strengthen safety claims.
(3) Success rates in simulated environments are described anecdotally. Including quantitative metrics would bolster robustness assertions.

Reviewer #5

(Remarks to the Author)
After reviewing the revised manuscript and the authors' detailed responses to the previous round of reviews, I am pleased to see that the authors have undertaken a comprehensive revision of the manuscript. They have effectively rewritten most of the content and conducted extensive supplementary experiments to address concerns regarding safety, system characterization, and comparative analysis. The manuscript is now technically sound and makes a clear contribution to the field of biohybrid robotics.

However, the current manuscript lacks a comprehensive examination of limitations and a corresponding discussion. Specifically, I have noted the following points:

1. The authors selected the Madagascar hissing cockroach (Gromphadorhina portentosa) as the experimental platform due to its superior characteristics, such as size and load-bearing capacity. However, this species has strong regional specificity and may not be the optimal choice for all potential deployment scenarios. To enhance the practical significance and broader impact of this work, I suggest the authors add a section to the Discussion regarding the potential challenges and coping strategies for developing similar "cyborg suits" for insects with different characteristics (e.g., smaller body sizes or different locomotive habits).

2. The current oxygen generation system operates passively; once the $H_2O_2$ is introduced to the $MnO_2$-deposited sponge, the reaction proceeds until the reagents are exhausted. While effective for the demonstrated duration, the manuscript would benefit from a brief discussion on future possibilities for active regulation. For instance, is it theoretically possible to incorporate micro-valves or separation mechanisms to control the reaction rate dynamically?

3. The methods describe sealing the flexible shell to the cockroach's first abdominal segment using a nitrile rubber membrane and adhesive to ensure waterproofing. Biologically, this permanent attachment would likely interfere with the insect’s natural molting process. While the focus of this study is appropriately on "hours-long" missions, it would be transparent to include a brief sentence in the discussion acknowledging this constraint. Clarifying that this system is intended for terminal deployment durations or inter-molt periods would improve the biological rigor of the study.

Version 2:

Reviewer comments:

Reviewer #2

(Remarks to the Author)
The authors have made high-quality revisions that have fully addressed the review comments. The manuscript is now significantly improved in both clarity and scientific rigor. Therefore, I recommend acceptance for publication.

Reviewer #5

(Remarks to the Author)
The authors addressed my comments quite well. I, therefore, recommend publication at Nature Communications.

**Open Access** This Peer Review File is licensed under a Creative Commons Attribution 4.0 International License, which permits use, sharing, adaptation, distribution and reproduction in any medium or format, as long as you give appropriate credit to the original author(s) and the source, provide a link to the Creative Commons license, and indicate if changes were made.
In cases where reviewers are anonymous, credit should be given to 'Anonymous Referee' and the source.
The images or other third party material in this Peer Review File are included in the article’s Creative Commons license, unless indicated otherwise in a credit line to the material. If material is not included in the article’s Creative Commons license and your intended use is not permitted by statutory regulation or exceeds the permitted use, you will need to obtain permission directly from the copyright holder.
To view a copy of this license, visit https://creativecommons.org/licenses/by/4.0/

# RESPONSES to REVIEWERS

Manuscript ID: NCOMMS-25-24554-T

Journal: Nature Communications

Dear Reviewers,

We sincerely thank you for your thoughtful, detailed, and constructive comments. In response, we have performed additional experiments and revised the manuscript accordingly.

A point-by-point response to all reviewer comments is provided on the following pages. **The original comments are presented in black font**, and **our responses in blue font**. For ease of reference, all modifications made to **the revised manuscript are also highlighted in blue font**.

We are deeply grateful for the reviewers' insightful guidance, which has substantially improved the clarity, rigor, and overall persuasiveness of the manuscript.

Thank you for your time and advice.

Sincerely,

All the authors represented by

Hirotaka Sato, Nanyang Technological University, Singapore

**REVIEWER COMMENTS**

**Reviewer #1:**

[R1-1] The paper "Underwater Suit-Wearing Cyborg Insect Capable of Hours-Long Diving and Terra-Aqua Travel" by Fan et al. presents a cyborg insect system where Madagascar hissing cockroaches are equipped with a wearable underwater suit that enables survival underwater for several hours. The suit features a 3D-printed shell and an oxygen generation system using $MnO_2$ and $H_2O_2$, aiming to support insect-based exploration in hazardous environments.

**[Reply to this comment]**

Thank you for taking the time to review our manuscript and for your insightful feedback. We have carefully reviewed your comments and conducted further investigations to address each of your concerns. In the following responses, we provide detailed explanations and modifications made to the manuscript. We believe these revisions have strengthened the manuscript.

[R1-2] The introduction reads more like a review and lacks a focused scientific question or hypothesis.

**[Reply to this comment]**

We thank the reviewer for this insightful suggestion. In the revised manuscript, we have restructured the introduction section to adopt a hypothesis-driven approach, emphasising novelty and significance:

a) Explicit hypothesis-driven added.

We hypothesize that by isolating cyborg insects from surrounding water while providing continuous oxygen supply, this insect-machine hybrid robot can achieve prolonged underwater survival.

b) Novelty and significance made explicit.

- Novelty: This work provides the first demonstration of an amphibious cyborg insect that can survives underwater for hours while preserving terrestrial locomotion.
- Significance: (i) Our design overcomes the physiological constraints that have previously limited biohybrid robots' operating environments. This allows functions confined to land to be extended into underwater settings, laying the foundation for field-deployable biohybrid systems in other extreme environments. (ii) Compared with conventional amphibious robots, our approach provides a flexible, low-power amphibious robot that preserves speed, manoeuvrability and is suitable for outdoor deployment in confined, cluttered terrestrial–aquatic environments.

**[Changes in manuscript]**

Section: 1. Introduction

Line 32 – 72 in manuscript.

[R1-3] No empirical data is provided on oxygen levels maintained inside the shell.

**[Reply to this comment]**

We have now included measurement of oxygen levels inside the suits (Fig. R1A). Besides, the oxygen generation rate and consumption rate was measured to estimate the survival time of cyborg cockroach. As a result, 1.0 ml $H_2O_2$ can produce 6.2 ± 0.3 ml oxygen (Fig. R1B), and the cyborg cockroach consumes 3.8 ± 0.6 ml/h (mean ± standard error, n = 12, N = 3) while walking. This indicates that cyborg cockroach equipped with suits can continue to walk over 1 hour.

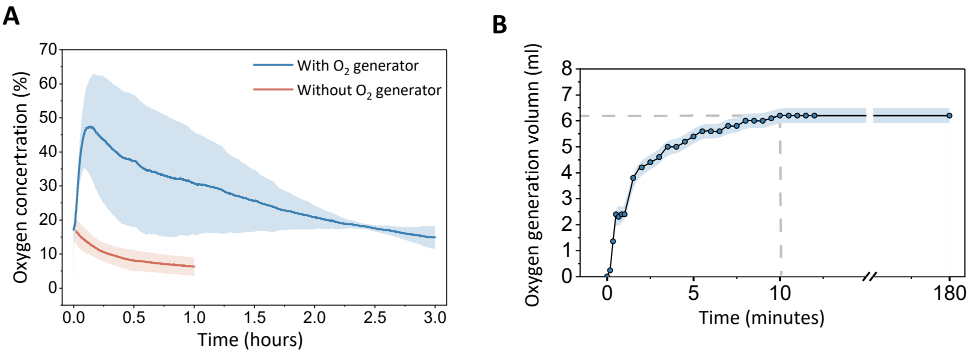


Figure R1: Evaluation of oxygen generation performance. (A) Oxygen level in the suits with and without oxygen generation system. Solid lines indicate the mean $O_2$ level, and the shaded area indicate the standard deviation. (B) Oxygen generation volume (mean ± standard deviation, n = 3).

**[Changes in manuscript]**

Section: 2.2 In-suit Oxygen Concentration Monitoring and Metabolic Rate

Line 185 – 186 in manuscript.

Line 188 – 196 in manuscript.

Line 207 – 212 in manuscript.

Section: 3.7 Measurement of Oxygen Generation Rate and Oxygen Consumption Rate

Line 429 – 440 in manuscript.

Fig. 3A, 3C.

Supplementary Fig. 6.

[R1-4] There's no mention of how long the cockroaches survived in water with and without the suit in controlled tests.

**[Reply to this comment]**

We agree reporting survival duration with and without the suit is indeed critical.

Accordingly, we conducted immersion experiments under identical conditions (same tank, water depth, and temperature) with continuous video recording. Cyborg insects equipped with diving suit survived for 3 h while remaining responsive and capable of following commanded movements (Supplementary Movie S1). By contrast, naive cockroach without diving suit became unresponsive and entered hypoxia underwater within approximately 2 min (we saved it by drying and moving it to fresh air, and it recovered fully within a few minutes) (Supplementary Movie S1). This comparison shows the effectiveness of the wearable diving suit in sustaining underwater survival.

**[Changes in manuscript]**

Section: 2.3 Survivability and Locomotion Test

Line 215 – 219 in manuscript.

Supplementary Video S1.

[R1-5] No comparison to existing solutions or systems is presented quantitatively.

**[Reply to this comment]**

To address this point, we have added a quantitative comparison of speed in both on-land and underwater with existing amphibious robots[1–15]. Specifically, we normalized the speed to body length per second (BL/s), as this dimensionless metric is widely used in robotics to enable fair comparison among systems of different scales. Similar normalization methods have been adopted in various studies[16–18]. The results show that the locomotion speed of cyborg insects is competitive both on land and underwater (Fig. R2). Most amphibious robots exhibit normalized speeds below 1.0 BL/s on land, whereas our system achieves 1.1 BL/s, comparable to or exceeding larger robots (Fig. R2A). Similarly, underwater speeds of most reported robots are below 0.8 BL/s, while our system reaches 0.9 BL/s, ranking among the higher-performing designs (Fig. R2B). This quantitative analysis demonstrate that our system performs competitively with or

above the level of reported designs.

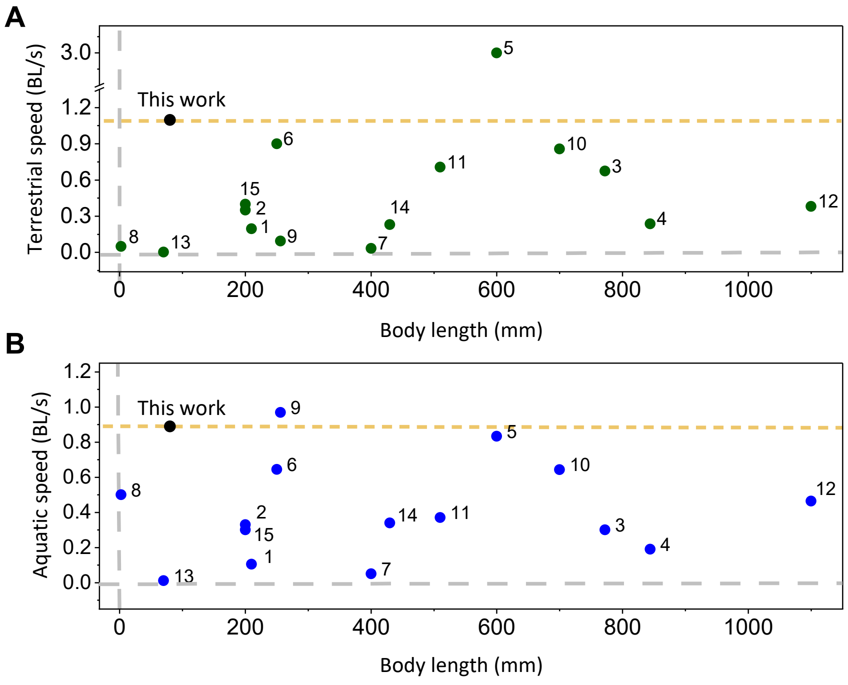


Figure R2: Locomotion performance comparison of the amphibious cyborg insects with other amphibious robots. Numbers correspond to the cited works in the references section. (A) Terrestrial speed comparison (B) Aquatic speed comparison.

**[Changes in manuscript]**

Section: 2.3 Survivability and Locomotion Test

Line 264 – 269 in manuscript.

Fig. 4C.

[R1-6] Has the suit been tested in simulated hazardous environments (e.g., smoky, flooded tunnels)?

**[Reply to this comment]**

Evaluating the suit in a mixed hazardous environment is indeed valuable. Following this idea, we constructed a tunnel system reproducing the reviewer-described conditions

and conducted traversal tests using cyborg insects. This allowed us to directly assess the suit's protective performance under combined gas-exposure and underwater conditions, as summarised below.

Specifically, we designed a $CO_2$–water tunnel system to simulate a mixed hazardous scenario combining anaesthetic gas exposure and water immersion (Fig. R3A). By comparing the behaviours of cyborg insects with and without the diving suit, the protective effectiveness of the suit was evaluated.

Cyborg insects without the suit immediately exhibited signs of distress following $CO_2$ injection, including disoriented movement, erratic leg movements, and loss of responsiveness to external stimuli within seconds (Fig. R3B, Supplementary Movie S3). These behaviours indicate that exposure to high concentrations of $CO_2$ rapidly induces anaesthesia, rendering the insect's incapable of traversing the $CO_2$ zone. After experiment, we saved the cockroach by moving it to fresh air, and it recovered fully within a few minutes. To further assess the effect of submersion, another cyborg insect without suit was placed directly before the water-filled zone. Upon receiving stimulation commands to walk into the water, it struggled and entered to a state of asphyxia within 45 s (Fig. R3C, Supplementary Movie S3). We saved it by moving it to fresh air, and it recovered fully within a few minutes

In contrast, the cyborg insect equipped with the diving suit maintained active locomotion throughout the experiment. It successfully traversed the $CO_2$ zone in approximately 50 s, entered the submerged section, and continued to move controllably underwater (Fig. R3D, Supplementary Movie S3). The insect subsequently emerged from the tunnel after a total duration of 2 min 45 s, demonstrating effective protection and sustained functionality in this mixed hazardous environment. Thank you for the idea of the test.

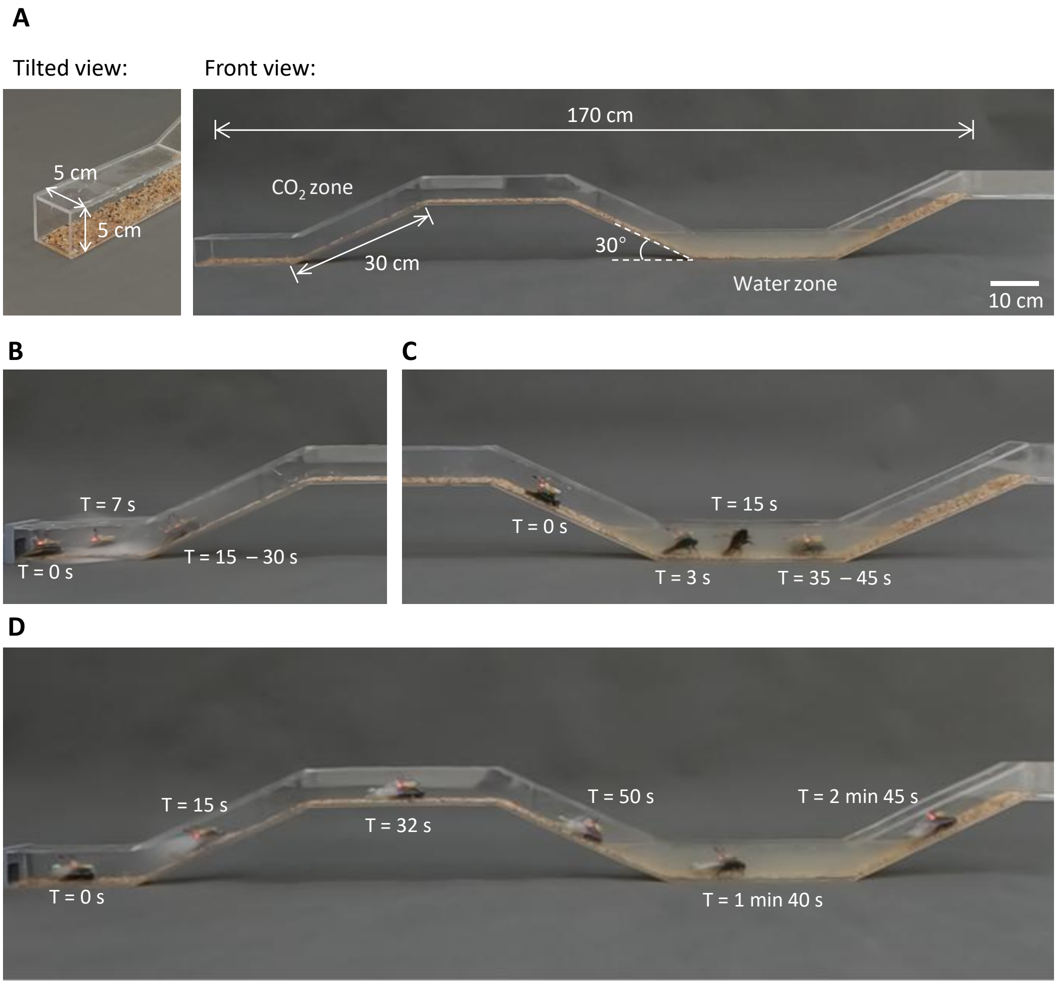


Figure R2: Comparison of locomotion performance of cyborg cockroaches with and without diving suit in simulated hazard environments. (A) Schematic of the tunnel setup with a length of 1.7 m and a cross-sectional size of 5 cm by 5 cm. The tunnel includes two regions: a $CO_2$ zone and a water zone. (B) Locomotion sequence of cyborg cockroach without suit in $CO_2$ zone. The cockroach was anaesthetized after $CO_2$ exposure (we saved it by moving it to fresh air, and it recovered fully within a few minutes). (C) Locomotion sequence of cyborg cockroach in underwater zone. The cockroach became asphyxiated 45 s after entering the water (we saved it by drying and moving it to fresh air, it recovered fully within a few minutes). (D) Locomotion sequence of cyborg cockroach in tunnel with $CO_2$ zone and underwater zone. The cockroach successfully passed through the entire tunnel in 2 min 45 s.

**[Changes in manuscript]**

Section: 2.4 Demonstrations of Survival, Adaptation, and Functional Extension

Line 278 – 295 in manuscript.

Section: 3.9.1 Mixed-hazard Tunnel Traversal

Line 455 – 462 in manuscript.

Fig. 5.

Supplementary Movie S3.

[R1-7] What metrics were used to assess system performance, endurance, and behaviour under stress? Without such validations, the claim of applicability in extreme environments remains speculative.

**[Reply to this comment]**

We thank the reviewer for raising this important concern regarding the metrics used to validate the system. The evaluation was conducted with metrics in three aspects:

a. System performance.

   We systematically quantified the locomotion performance of the wearable diving suit's biomimetic insect by measuring forward and angular speeds at multiple time points during prolonged underwater operations (5 min, 30 min, 1 h, 2 h, and 3 h), as shown in Figure R4. During extended continuous experiments, although the cyborg cockroach's locomotive speed is influenced by fatigue and stimulus habituation[19–21], it showed stable forward and turning behaviour at all test time points. Under electrical stimulation, both forward and angular speeds showed significant increase, indicating that command-responsive locomotor control remained effective throughout the 3-hour immersion period. The forward speed diminished from 71.1 mm/s at 5 min to 44.1 mm/s after 3 h. The right-turning

angular speed decreased from 29.8°/s to 10.7°/s, while the left-turning angular speed dropped from 34.9°/s to 14.1°/s. By comparison, previous study achieved long-term navigation of a cyborg cockroach in terrestrial environments[22]. With a forward speed of approximately 29.0 mm/s and angular speed of about 6.95°/s, precise point-to-point path control was realised. In contrast, the diving suit wearing cyborg insects maintains higher forward and angular speeds underwater, demonstrating its capacity for stable and precise command response and displacement control even underwater.

b. Endurance.

To assess robustness against mechanical shock, we conducted drop tests following the MIL-STD-810H standard, a test protocol widely used to evaluate reliability under harsh conditions. The MIL-STD-810H protocol defines multi-orientation drop tests to assess robustness under mechanical shock. Following this principle, we tested the suit-wearing cyborg insect by dropping it from 20 cm to 1 m at different orientations. In all cases, the diving suit remained intact without structural fracture, the oxygen generator continued to operate without leakage, and the cyborg insects remained active. These outcomes demonstrate that the system withstands mechanical shocks comparable to field-level accidental impacts.

c. Behaviour under stress.

To further evaluate performance under hydrostatic stress, we immersed the cyborg insects in a container where the water depth was sequentially increased from 5 to 50 cm (≈ 20 body height). At each depth, the cyborg insects were maintained for 5 minutes. At all depths, the underwater suit retained structural integrity, no leakage of $H_2O_2$ was observed, and the insect was survived and maintained locomotion. These results demonstrate that the system is robust against increased water pressure and continues to function effectively under demanding environmental stress.

Together, these validations show that the system preserves locomotion, structural robustness, and physiological stability under diverse extreme conditions, substantiating its applicability in challenging environments.

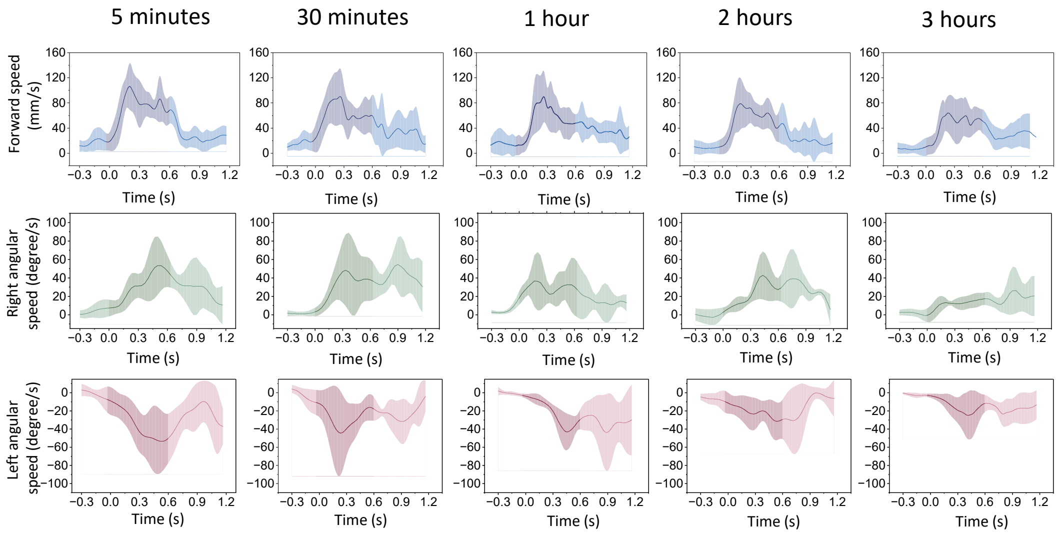


Figure R3: Underwater speed at multiple time points after immersion (mean ± standard deviation).

**[Changes in manuscript]**

Section: 2.1 Integrative Design of Diving Suit for Underwater Survival and Mobility

Line 163 – 175 in manuscript.

Section: 2.3 Survivability and Locomotion Test

Line 239 – 248 in manuscript.

Fig. 4D.

[R1-8] Using $MnO_2$ and $H_2O_2$ in proximity to living organisms raises significant concerns regarding toxicity, heat generation, and potential harm to the insect. No discussion is provided on the possible biological impact of long-term exposure to reaction byproducts. The environmental safety of deploying such cyborgs with chemical tanks in natural disaster zones is unaddressed.

**[Reply to this comment]**

Thank you for your insightful comment. Your concerns helped us realize that the

original design did indeed have potential risks, such as leakage when cockroach is tilted or overturned, which could cause $H_2O_2$ to leak from the oxygen generator. To address these issues, we have redesigned the oxygen generator and address safety on four directions: (a) containment and leakage prevention; (b) thermal management; (c) reagent toxicity and potential long-term exposure to reaction byproducts; and (d) environmental safety for field deployment.

a. Containment and leak prevention.

   The new oxygen generator is a sealed container (Fig. R5A), releasing oxygen exclusively through a hydrophobic PTFE microporous membrane with a pore size of 0.22 μm (Fig. R5B). The $H_2O_2$ solution and $MnO_2$-deposited sponge are confined within the container, and the lid–reservoir interface is sealed with UV-curable adhesive. The sealing stability of the oxygen generator was evaluated by subjecting the assembled unit to agitation on a vortex shaker for 10 minutes to simulate mechanical shocks. Following this, the lid surface was wiped with water-sensitive test paper, with no colour change observed. The unit was then positioned with the lid and membrane facing downwards over another piece of water-sensitive test paper for 3 h. No colour change was detected, indicating that no liquid leakage had occurred.

b. Thermal management.

   In our oxygen generation system, only small reagent loads were used (only 2.0 mg $MnO_2$ deposited onto the sponge, with the added 1.0 ml of 3% $H_2O_2$), ensuring a very low reaction rate. An infrared camera (Ti400, Fluke) showed the surface temperature remaining between 23.6 and 24.0°C throughout the reaction (Fig. R5C), confirming negligible heat generation under these conditions.

c. Reagent toxicity and long-term exposure to byproducts.

   The catalytic reaction produces only oxygen (vented) and water (retained in the generator), with no additional volatile byproducts. Reagent loads and concentrations are low. Both $MnO_2$ powders and the $H_2O_2$ solution remain confined within the generator and do not contact the insect. Across repeated trials involving

multiple insects, we observed no observable adverse physiological or behavioural effects, indicating minimal risk of long-term toxicity.

d. Environmental safety for field use.

The system is designed for no-release operation: reagents remain fully contained, and residual liquids are retained in the generator. Only oxygen diffuses through the membrane into the interior of the shell to support respiration. The suit itself is sealed and does not exchange gas with the external environment; both generated $O_2$ and exhaled $CO_2$ remain within the suit during underwater operation.

Together, the modifications can ensure the safety of the insects, minimize the toxicological risks, and support the environmental suitability of the system for potential field deployment.

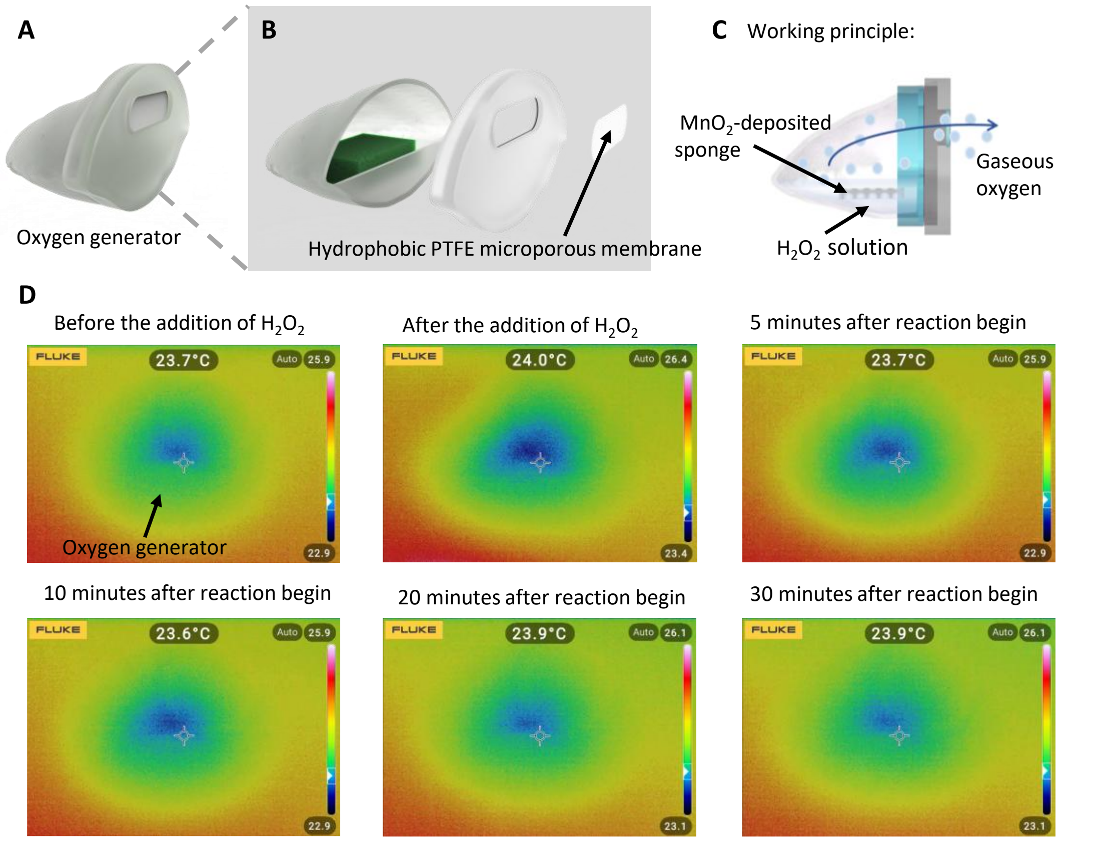


Figure R4: Design of the Oxygen generator. (A) 3D schematic of the sealed oxygen generator. (B) Exploded view of the oxygen generator. It includes: a container with a $MnO_2$-deposited cellulose sponge inside, a sealing lid and a hydrophobic PTFE

microporous membrane. (C) Working principle of hydrophobic PTFE microporous membrane. It allows gases to pass through its micropores while blocking liquid.

**[Changes in manuscript]**

Section: 2.1 Integrative Design of Diving Suit for Underwater Survival and Mobility

Line 83 – 85 in manuscript.

Line 93 – 116 in manuscript.

Fig. 2B.

Supplementary Fig. S2.

[R1-9] The manuscript suffers from repetitive phrasing, unclear transitions, and poor figure referencing (e.g., “Fig. S1” appears without prior context). Additionally, the structure lacks coherence in moving from background to methodology to result interpretation. Critical technical details are buried within long paragraphs without proper emphasis or figure cross-referencing.

**[Reply to this comment]**

In the revised manuscript, we have streamlined the text, removed redundant statements and improved transitions between sections. We have ensured all figures are correctly referenced. These revisions significantly enhance readability, logical coherence, and the clarity of figure cross-referencing. Thank you for your advice.

[R1-10] English needs a deep revision.

**[Reply to this comment]**

In the revised manuscript, we have significantly improved the clarity and overall readability of the English. The text has been revised with input from native English

speakers, and we also commissioned a professional proofreading service from Wordvice to address grammatical and typographical issues throughout the manuscript. The corresponding proofreading certificate is included at the end of this response letter. Thank you for your suggestion.

**Reviewer #2:**

[R2-1] This study presents an innovative biohybrid system combining a Madagascar hissing cockroach with a wearable underwater suit, enabling prolonged amphibious operation. The integration of an oxygen supply system ($MnO_2$-$H_2O_2$ reaction) and a 3D-printed flexible shell demonstrates potential for search-and-rescue and environmental monitoring applications. While the work is groundbreaking, major revisions are required to address experimental gaps. Below are detailed critiques and recommendations.

**[Reply to this comment]**

Thank you very much for your enlightening advice. We have learned a lot from your comments. Based on your suggestions, quality of our manuscripts has been improved significantly. We have responded to each of your comments below.

[R2-2] Oxygen Supply System: Insect locomotion as well as energy consumption tends to be stronger in the electrically stimulated state than in the unstimulated state. Therefore, the $MnO_2$-$H_2O_2$ reaction rates should be correlated with the cockroach's metabolic demand across different activity states (with or without electrical stimulation). Plot oxygen consumption vs. generation rates dynamically.

**[Reply to this comment]**

We agree that the energy consumption of insect varies depending on the state of activity. The current suits design does not include dynamic $H_2O_2$ delivery system, leading to sudden increase of oxygen level followed by monotonic decrease due to consumption by insect (e.g., Fig. 3C in revised manuscript). However, the oxygen level in the suits was still within the range where insects can survive for days and maintain their locomotor performance. Insects exhibit notable adaptability to hypoxia as well as hyperoxia, which insects are unlikely to encounter in the wild. For example, various insects can grow in wide range of oxygen level (cockroach[23]: 12% - 31%, moth [24]: 10%

- 40%, beetle [25]: 10% - 40%). Honeybee[26] and dragonfly[27] are able to fly in atmosphere containing 10% - 40% $O_2$. The previous study in *Drosophila*[28] showed that 90% of the population was surviving ($LS_{90}$: considered as healthy lifespan) at 5% - 80% $O_2$ level for 10 days and that flies reared up to 60% $O_2$ level exhibited the climbing performance comparable to those in normoxia for 5 days. Therefore, the oxygen supply system is considered suitable for cyborg insect, capable of sustaining oxygen requirements for at least several hours. Meanwhile, the previous manuscript did not provide any data to show if the oxygen supply was sufficient or not. To address this, we have measured the oxygen production rate and consumption rate. As a result, 1.0 ml $H_2O_2$ can produce 6.2 ± 0.3 ml oxygen, and the cyborg cockroach consumes 3.8 ± 0.6 ml/h (mean ± standard error, n = 12, N = 3) while walking. This indicates that cyborg cockroach equipped with suits can continue to walk over 1 hour by adding 1.0 ml of $H_2O_2$. These results indicate that supplying 1.0 ml of $H_2O_2$ is sufficient to support walking for over one hour. We sincerely thank the reviewer for raising this point, which allowed us to strengthen the discussion of the oxygen supply system.

**[Change in manuscript]**

Section: 2.2 In-suit Oxygen Concentration Monitoring and Metabolic Rate

Line 185 – 186 in manuscript.

Line 207 – 212 in manuscript.

Section: 3.7 Measurement of Oxygen Generation Rate and Oxygen Consumption Rate

Line 429 – 440 in manuscript.

Fig. 3A

Supplementary Fig. 6.

[R2-3] Wearable underwater suit: Although the morphology of the underwater suit was designed with reference to the real form of the cockroach abdomen, did the design of the dimensions take into account the effects on the cockroach's locomotor behaviour and the deformation of the abdomen?

**[Reply to this comment]**

Thank you for the question. During suit design, we considered not only the real form of the cockroach abdomen but also the potential impact of the diving suit on locomotor behaviour and abdominal deformation.

a. Locomotor behaviour:

   Previous studies compared the speed and success rate of cockroaches traversing complex environments while wearing different shell types (oval cone, flat oval, and flat rectangle)[29]. Results showed that the oval cone shell significantly outperformed the other two forms in both speed and success rate. This finding indicates that rounded, streamlined shell shape enable cockroaches to navigate complex environments more smoothly while reducing snagging incidents.

   Base on this result, we incorporated a near-circular/elliptical cross-sectional structure into the diving suit design to minimize the impact of air resistance and water flow resistance on cyborg insects during both terrestrial and aquatic movement. Additionally, the suit's length exceeds the maximum abdominal extension measured during cockroach locomotion, with a space allowance ensuring it does not disturb with natural leg swinging during movement. These design optimizations enable the suit to provide protective functionality while maximally preserving the cockroach's natural locomotor behaviour.

b. Abdominal deformation

   The deformation of a cockroach's abdomen involves respiratory contractions and swinging motions during locomotion such as turning or climbing. In our design, the diving suit uses an extra soft rubber membrane as the interface connecting to cockroach’s first abdomen segment (Fig. R6A). The membrane itself served only

as a waterproof barrier and connection layer without imposing any force or mechanical constraints. This compliant connection accommodates abdominal movements without imposing any restrictions or forces on them (Fig. R6B–D). This ensures both respiratory contractions and movement-induced bending are preserved. Consequently, the suit's impact on the insect's natural abdominal deformation and locomotive performance remains within acceptable limits.

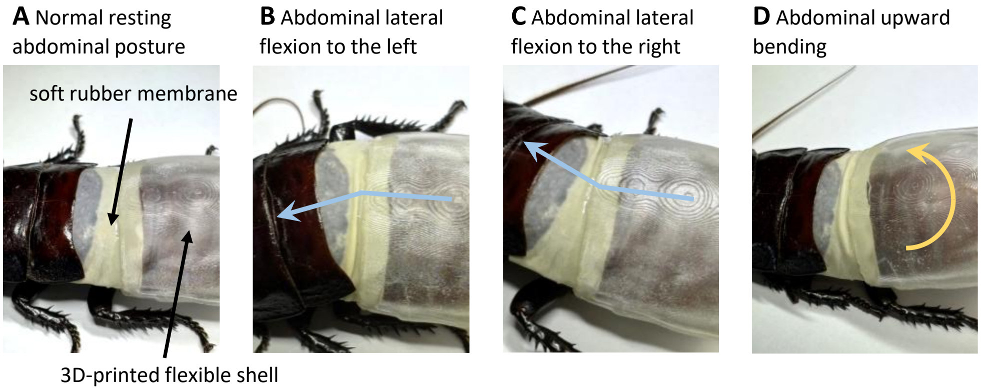


Figure R5: Abdominal mobility of cyborg insects with the wearable diving suit. (A) Normal resting posture. (B–C) Lateral flexion to the left and right. (D) Upward bending of the abdomen.

**[Changes in manuscript]**

Section: 2.1 Integrative Design of Diving Suit for Underwater Survival and Mobility

Line 129 – 140 in manuscript.

Supplementary Fig. S3.

[R2-4] Wearable underwater suit: The risk of catalyst failure or liquid leakage during the operation of cockroaches underwater has not been evaluated.

**[Reply to this comment]**

Thank you for your feedback. We realized the potential risks in our original design,

particularly concerning the possibility of $H_2O_2$ leakage when the cockroach tilt or overturn. To address this, we have undertaken a comprehensive redesign of the oxygen generator, eliminating the risk of catalyst failure or liquid leakage entirely while maintaining its functionality.

a. Leakage risk

The new tank design has a double barrier (UV-curable adhesive bond and mechanical fit) at the lid–reservoir interface (Fig. R7A). A hydrophobic PTFE microporous membrane with a pore size of 0.22 μm was used as the only gas outlet (Fig. R7B). All liquid reagents are confined within the reservoir. Only the generated oxygen in gas state can be released from the oxygen generator. To assess the sealing performance of the oxygen generator, the assembled unit was subjected to mechanical shock simulation by being agitated on a vortex shaker for 10 min. Following this, the lid was wiped with water-sensitive test paper, and no colour change was observed, indicating no leakage. The unit was then placed with the lid and membrane facing down on another piece of water-sensitive test paper for a 3-hour period. After this duration, the paper showed no signs of colour change, confirming that no liquid had leaked from the unit.

b. Catalyst failure

In our new design, $MnO_2$ and $H_2O_2$ are co-located within a sealed generator, with only oxygen can exit through the hydrophobic PTFE membrane; this architecture ensures continuous reactant contact and precludes failure modes arising from separation. Potential deactivation environments for the $MnO_2$–$H_2O_2$ system include acidic pH that promotes Mn dissolution, elevated ionic strength, phosphate or silicate contamination that poisons active sites, surface passivation by adsorbates, and thermal stress[30,31]. Our experiments explicitly exclude these conditions. We maintain near-neutral pH with deionized water and low ionic strength; reagents are phosphate- and silicate-free; $MnO_2$ is immobilized within a cellulose sponge to prevent migration and agglomeration; 3% $H_2O_2$ is used at low dose; and all tests are performed at ambient temperature. These limiting

factors preclude the possibility of catalytic deactivation.

Across all underwater experiments, we have observed neither catalyst failure nor liquid leakage. Together, these design choices and empirical checks indicate a low probability of oxygen generation failure under our operating conditions.

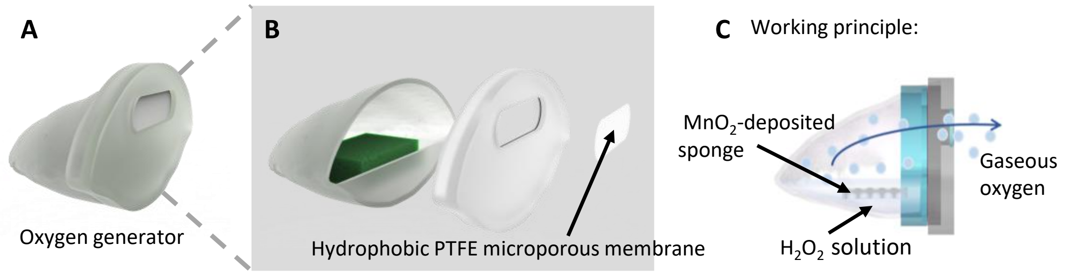


Figure R6: Design of the Oxygen generator. (A) 3D schematic of the sealed oxygen generator. (B) Exploded view of the oxygen generator. It includes: a container with a $MnO_2$-deposited cellulose sponge inside, a sealing lid and a hydrophobic PTFE microporous membrane. (C) Working principle of hydrophobic PTFE microporous membrane. It allows gases to pass through its micropores while blocking liquid.

**[Changes in manuscript]**

Section: 2.1 Integrative Design of Diving Suit for Underwater Survival and Mobility

Line 83 – 85 in manuscript.

Line 93 – 116 in manuscript.

Fig. 2B.

[R2-5] Wearable underwater suit: Durability experiments on the sealing of waterproof clothing underwater need to be increased.

**[Reply to this comment]**

To verify waterproof sealing, we conducted tests by placing water-sensitive test paper inside the diving suit (Fig. R8A). The cyborg cockroach with diving suit was submerged

in water for 30 min and experienced mechanical bending. After testing, the water-sensitive paper retrieved from the suit exhibited only a slight colour difference compared to its original yellow colour before the experiment, with no significant blue change indicating water penetration (Fig. R8A).

To further clarify the source of this slight change, we conducted two control experiments. First, when the paper was placed inside the suit while the cockroach remained on land for 30 min, the paper exhibited a similar slight discoloration (Fig. R8B, i). This result indicates the minor colour difference originated from moisture generated by the insect's respiration rather than water penetration. Second, we compared control paper samples exposed to humid air and direct water contact. Paper exposed to humid air turned deep blue, while paper in direct water contact turned blue and become faded (Fig. R8B, ii-iii). In contrast, test paper placed inside the diving suit after underwater immersion showed small colour difference compared to the terrestrial result and its original colour, with its colour change consistently less pronounced than that observed with direct water contact.

Together, these experiments confirm that no water entered the suit during the underwater durability test. The waterproof sealing remained intact and durable throughout repeated submersion and bending, thereby validating the effectiveness and robustness of the suit design.

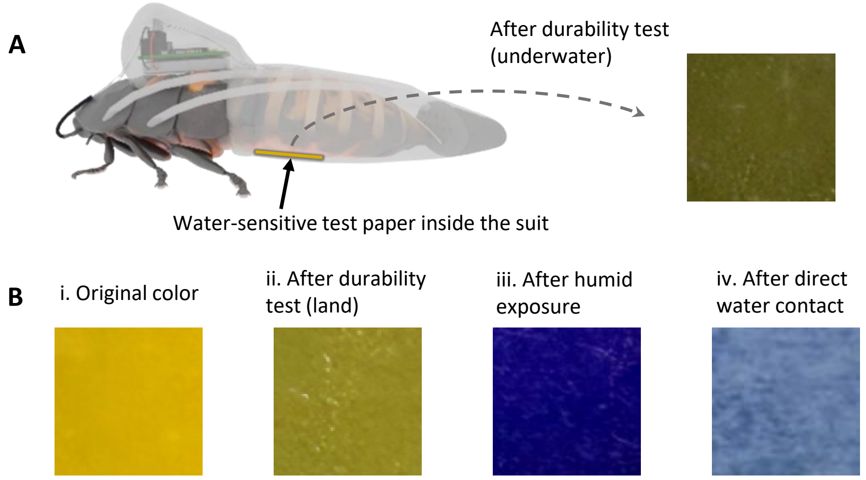


Figure R7: Waterproof performance of the diving suit evaluated with water-sensitive test paper. (A) Placement of water-sensitive paper inside the suit during immersion testing. After underwater durability testing, the paper maintained its original yellowish colour, indicating no water infiltration. (B) Reference colour changes of the test paper under different conditions. i) original colour test paper. ii) test paper placed inside the suit on land. iii) test paper exposed to a humid environment. iv) test paper directly contacted with water.

**[Changes in manuscript]**

Section: 2.1 Integrative Design of Diving Suit for Underwater Survival and Mobility

Line 155 – 163 in manuscript.

Section: 3.5 Evaluation of Waterproof under Immersion and Bending

Line 388 – 399 in manuscript.

Supplementary Fig. S4.

[R2-6] Locomotion control: Although the authors mentioned the motion attenuation of cockroaches under water. It is suggested to further explore how to improve the speed and stability of cockroach's movement in underwater environments and how to extend its task execution time.

**[Reply to this comment]**

We thank the reviewer for this constructive suggestion. In our current design, we have already considered several factors to mitigate motion attenuation in underwater environments. Specifically, the morphology, weight, and buoyancy distribution of the suit were carefully optimized to enhance locomotion stability, and we have also conducted preliminary attempts to adjust stimulation patterns to sustain underwater movement.

For future improvement, we will further investigate the following aspects in more detail:

a. Optimization of stimulation parameters: We will systematically vary the stimulation frequency, intensity, and duration. Previous studies indicate that cockroaches tend to become less responsive to repetitive stimuli over time[19,21,22]. However, in the current measurement of underwater locomotion speed, we deliberately applied the same stimulation voltage and duration across all tests to ensure experimental consistency and comparability of the results. Inspired by the adaptive stimulation algorithm proposed by Li et al.[20], which dynamically adjusts stimulation voltage based on real-time motion feedback to counteract adaptive attenuation, this system may incorporate a similar feedback-regulated control scheme. This approach holds promise for sustaining responsiveness, mitigating motion decay, and maintaining stable locomotion during prolonged underwater operations.

b. Suit design modifications: We intend to further optimize the design of the suit. One approach is to integrate a small omni-directional wheel at the bottom of the suit instead of a counterweight. This modification is expected to both counteract buoyancy and reduce ground friction, thereby facilitating smoother forward movement and reducing energy consumption by the insect. Following preliminary

testing, we have observed that the cyborg insects with newly designed suit achieves an increase in speed compared to the original one.

c. Extension of operational time: The duration of underwater locomotion is currently limited by the supply of oxygen generated from the $MnO_2$–$H_2O_2$ reaction. Increasing the concentration or volume of $H_2O_2$ can increase oxygen generation, enabling the system to support longer durations of underwater locomotion.

These strategies provide feasible solutions for future optimization.

[R2-7] Additional Weaknesses: The manuscript lacks quantitative comparisons with existing amphibious robots.

**[Reply to this comment]**

We have added quantitative comparisons with reported amphibious robots in terms of speed and payload capacity.

a. Speed comparison:

Cyborg insects with diving suit have an average terrestrial speed of 87.7 mm/s and average aquatic speed of 71.1 mm/s. To enable direct comparison between robots of varying scales, speed values were standardised relative to body length (BL/s). This constitutes the standard practice for evaluating the locomotive performance of animals and robots of differing dimensions. Within the field of robotic systems, BL/s remains the consistently adopted metric for assessment across numerous studies[16–18]. As shown in Figure R9, we reported data of existing amphibious robots operating on both land and underwater[1–15].

- On land, most reported robots show normalized speeds below 1.0 BL/s. Our cyborg insect system reaches 1.1 BL/s, which is competitive or superior compared with many existing robots.
- Underwater, most reported speeds fall below 0.8 BL/s, while our system reaches 0.9 BL/s, placing it among the higher-performing designs.

These quantitative comparisons demonstrate that our system achieves competitive locomotion relative to existing amphibious robots.

b. Payload capacity

The total load capacity of the cyborg cockroach is about 15 g. After wearing the 5.5 ± 0.3 g diving suit, the cyborg insect still retains an available payload of approximately 9.5 g. This payload is at the upper end of the range reported for centimetre-scale amphibious robots[32–35] and provides sufficient capacity for integrating additional functional modules such as cameras, sensors, and communication devices.

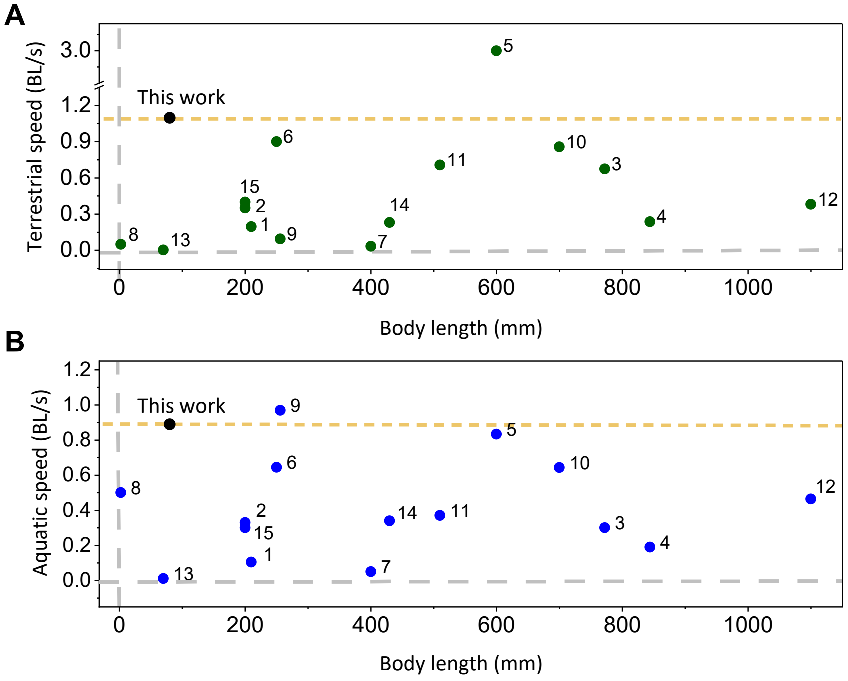


Figure R9: Locomotion performance comparison of the amphibious cyborg insects with other amphibious robots. Numbers correspond to the cited works in the references section. (A) Terrestrial speed comparison (B) Aquatic speed comparison.

**[Changes in manuscript]**

Section: 2.3 Survivability and Locomotion Test

Line 264 – 269 in manuscript.

Section: 3.3 Miniature Wireless Stimulator (Backpack)

Line 345 – 350 in manuscript.

Fig. 4C.

[R2-8] Additional Weaknesses: The introduction of experimental methods in Result and Discussion section is redundant and streamlining is recommended.

**[Reply to this comment]**

In the revised manuscript, we have streamlined this part by removing repetitive methodological details and retaining only the information necessary to support the interpretation of results. This restructuring makes the Results and Discussion section more concise and focused. Thank you for pointing this out.

**Reviewer #3:**

[R3-1] This manuscript presents an innovative system enabling amphibious capabilities in cyborg insects using a wearable underwater suit that integrates an oxygen generation system. The authors demonstrate that Madagascar hissing cockroaches can survive and maneuver underwater while carrying control and sensing modules. The concept is novel, and the experiments are generally well described. However, several claims need stronger experimental support, and further clarification is needed in multiple areas.

**[Reply to this comment]**

Thank you for time on reviewing our manuscript and providing insightful comments. In this response, we address your questions point-by-point and have revised the manuscript accordingly. We hope you find the revised manuscript to be clearer and more convincing.

[R3-2] The authors state that the cyborg insects "can operate effectively on land while also surviving underwater for several hours." However, the main data presented focuses on underwater operation. Please clarify whether there is experimental evidence that the insects with the suit and control systems can still navigate on land. If such data exists, please include it; if not, the claim should be rephrased.

**[Reply to this comment]**

We have experimental evidence confirming that the cyborg insects equipped with diving suit and control system can still navigate effectively on land. Specifically, we conducted stimulation experiments on land using the same setup as in underwater trials and measured locomotion data (Fig. R10). The average forward speed during stimulation on land was 87.7 ± 47.5 mm/s, with left-turning angular speed of 58.3 ± 41.2°/s and right-turning angular speed of 65.1 ± 37.4°/s.

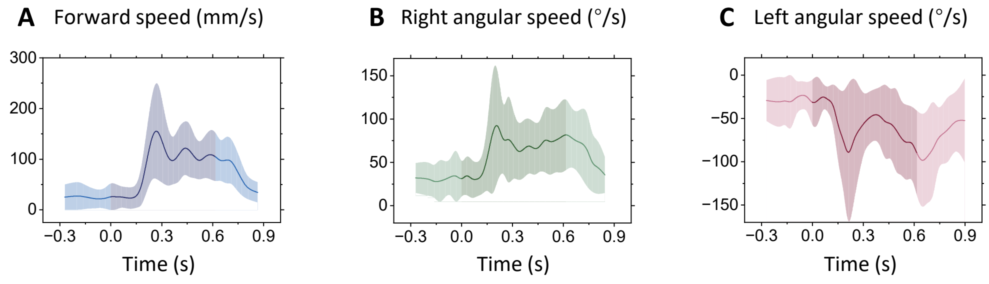


Figure R8: Locomotion speed of cyborg insects with diving suit on land. (A) Forward speed (mean ± SD). The average forward speed during the stimulation was 87.7 mm/s. (B) Angular speed during right turning (mean ± SD). The average angular speed during the stimulation was 65.1°/s. (C) Angular speed during left turning (mean ± SD). The average angular speed during the stimulation was 58.3°/s.

**[Changes in manuscript]**

Section: 2.3 Survivability and Locomotion Test

Line 225 – 228 in manuscript.

Fig. 4B.

[R3-3] The title references "hours-long diving." The data in Figure 2c and 2d show oxygen concentration and movement lasting up to 3 hours. Please confirm whether these data represent continuous, active underwater navigation and clarify what tasks were performed during that period. This would better support the claim.

**[Reply to this comment]**

Sorry for misleading. Figures 2C and 2D (in previous manuscript) were obtained under air conditions. The experimental setup is illustrated in Fig. R11B. In the experiments, we put the cyborg insects in sealed diving suits and conduct periodic electrical stimulation over hours to confirm their viability within the suit's self-sustaining oxygen environment. We have included actual walking period in the revised manuscript.

Besides, we have measured the oxygen production rate and consumption rate. As a result, 1.0 ml $H_2O_2$ can produce 6.2 ± 0.3 ml oxygen (Fig. R11A), and the cyborg cockroach consumes 3.8 ± 0.6 ml/h (mean ± standard error, n = 12, N =3) while walking. This indicates that cyborg insects equipped with diving suits can sustain movement for over an hour before oxygen depletion, and survive for 2–3 h under intermittent motion.

To verify whether the cyborg insect could survive and locomote underwater, we conducted additional experiment. Video recordings were captured over 3 h while the cyborg insects remained underwater and react to periodic stimulation (Supplementary Video S1). Results demonstrated that the insect retained its locomotor responsiveness throughout the entire three-hour underwater period, consistent with oxygen supply data obtained under air conditions. Thank you for the comment.

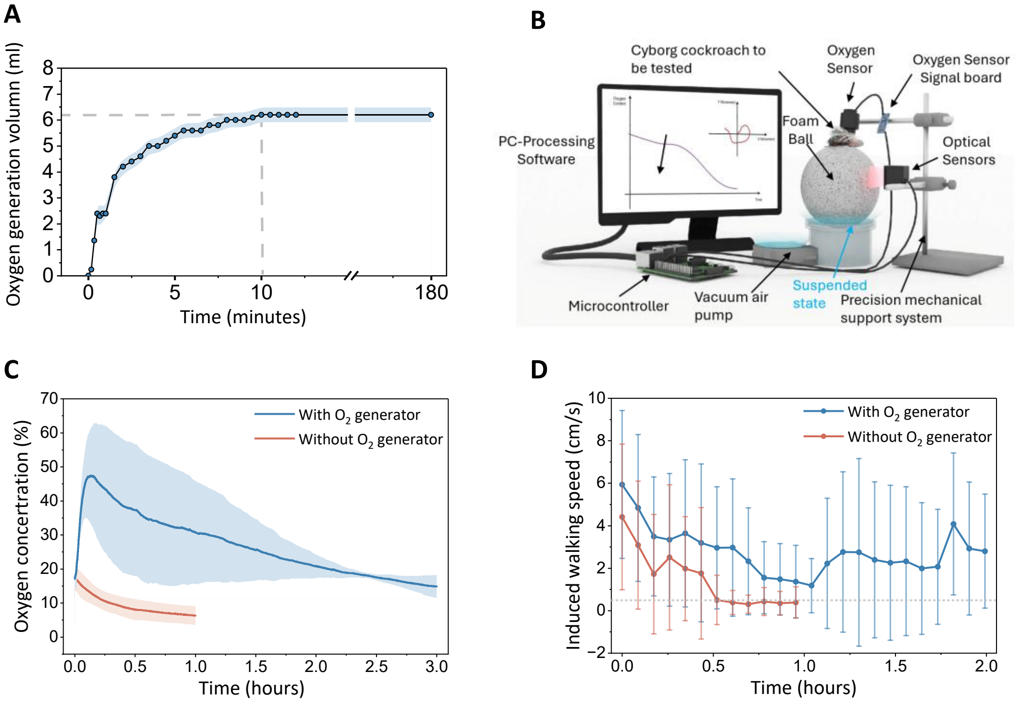


Figure R9. Oxygen level in the suits and locomotor performance. (A) Oxygen generation volume. (B) Treadmill system for measuring oxygen level and activity of cyborg cockroach. (C) Oxygen level in the suits with and without oxygen generation system. Solid lines indicate the mean $O_2$ level, and the shaded area indicate the standard

deviation. (D) Locomotor activity of cockroach. The thick lines indicate the mean value, and vertical lines indicate the standard deviation. The horizontal dotted line presents a walking speed of 0.5 cm/s.

**[Changes in manuscript]**

Section: 2.2 In-suit Oxygen Concentration Monitoring and Metabolic Rate

Line 185 – 186 in manuscript.

Line 205 – 212 in manuscript.

Section: 2.3 Survivability and Locomotion Test

Line 215 – 218 in manuscript.

Section: 3.7 Measurement of Oxygen Generation Rate and Oxygen Consumption Rate

Line 429 – 440 in manuscript.

Fig. 3A.

Supplementary Fig. 6.

[R3-4] A comparison with the state of the art is missing. Please contextualize the performance of your system in terms of speed and manoeuvrability --- both in air and water --- relative to other cyborg insect systems or miniaturized amphibious robots.

**[Reply to this comment]**

We have included a clearer contextualization of our system's performance relative to existing work.

a. Speed comparison

   We report locomotion speeds of amphibious robots both on land and underwater[1–15]. The speeds are normalized to body length per second (BL/s), as shown in Figure R12. On land, most miniature amphibious robots move at speeds below 1.0 BL/s,

while our cyborg insect system achieves 1.1 BL/s, placing it at the upper end of the reported range. In underwater conditions, most amphibious robots exhibit speeds under 0.8 BL/s. Our system reaches 0.9 BL/s, which is among the higher values reported for all amphibious robots. These comparisons demonstrate that our design provides efficient locomotion performance relative to other robot systems, despite its biological integration.

b. Manoeuvrability comparison

To assess the impact of the diving suit on the manoeuvrability of the cyborg cockroach, we first compared the angular speed of the cyborg cockroach with and without the diving suit. On land, the angular speed of the cyborg cockroach with diving suit reaches 58.3°/s for left turns and 65.1°/s for right turns. In underwater condition, the angular speed reaches 39.9°/s for left turns and 29.8°/s for right turns. In contrast, the angular speed without the diving suit typically ranges from 20 to 60°/s[36], and the cyborg cockroach with the diving suit maintains its angular speed within this range. This indicates that the design of the diving suit has minimal impact on manoeuvrability of a cyborg cockroach. When compared to other centimetre-scale amphibious robots with specified angular speeds[14,37,38], the cyborg cockroach with diving suit demonstrates several times higher angular speed on both land and underwater.

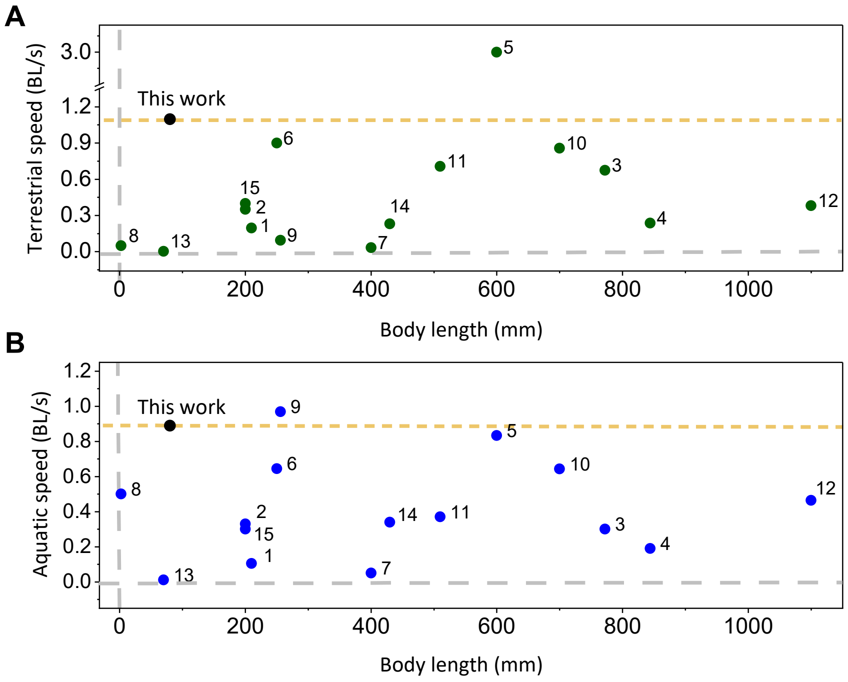


Figure R10. Locomotion performance comparison of the amphibious cyborg insects with other amphibious robots. Numbers correspond to the cited works in the references section. (A) Terrestrial speed comparison (B) Aquatic speed comparison.

**[Changes in manuscript]**

Section: 2.3 Survivability and Locomotion Test

Line 225 – 230 in manuscript.

Line 264 – 271 in manuscript.

Fig. 4C.

[R3-5] The manuscript does not mention how reliably the system works across trials. What was the success rate for underwater operation? Were there any observed failure modes such as water leakage or system malfunctions? Please comment on robustness.

**[Reply to this comment]**

We would like to provide the following additional information:

a. Failure modes

Some failure modes were identified and resolved at various stages. During the surgical implantation phase, instances occurred where excessive fixation of tubing resulted in partial obstruction of the spiracles. Such cases were excluded from subsequent experiments. During pre-operation checks, all components underwent microscopic inspection to verify the integrity of tube-to-spiracle connections and suit interfaces, followed by short-duration immersion leak tests to ensure complete sealing. Any sample failing inspection was reworked and excluded from formal operation. During the operational phase, an early failure mode manifested as leakage of $H_2O_2$ from the oxygen generator into the abdominal shell. While this did not cause immediate behavioural changes, it resulted in interrupted oxygen generation and reduced underwater operational duration. Since then, we have upgraded the design of the oxygen generator to completely eliminate the risk of leakage. No further failures related to $H_2O_2$ leakage or other system malfunctions have been observed during subsequent trials.

b. Success Rate:

Following the implementation of pre-operative inspection procedures and the upgrade of the oxygen generator, all subsequent trials demonstrated stable operational performance. No further malfunctions or anomalies occurred. Under optimized configurations, the cyborg insect reliably maintained normal respiration and command-responsive motor capabilities throughout continuous underwater operation exceeding 2 h. This indicates that the current system design and testing protocols effectively ensure long-term stable operation.

c. Robustness

Several design features were implemented to enhance the robustness of the system. These include multi-layer seals around the suit's installation gaps and a liquid leak-proof design of the oxygen tank, ensuring that the possibility of reaction failure due to leaks is eliminated. Additionally, we tested the system under varying water levels,

from 5 cm to 50 cm. In each of these levels, the system performed consistently, with the cockroach's behaviour remaining stable and the system operating normally. Furthermore, we performed drop tests from heights ranging from 20 cm to 1 meter, and the system's structure showed no signs of damage. These tests confirm the robustness of the system under different stress conditions.

**[Changes in manuscript]**

Section: 2.1 Integrative Design of Diving Suit for Underwater Survival and Mobility

Line 163 – 175 in manuscript.

Section: 3.4 Preparation of Diving Suit-Wearing Cyborg Cockroaches

Line 379 – 381 in manuscript.

[R3-6] Lines 122–136 discuss the $MnO_2$-$H_2O_2$ oxygen generation system. While the duration of oxygen generation is compared to metabolic demands, the actual oxygen volumes generated and consumed are not clearly matched. Please include these data to support the claim that oxygen generation was sufficient.

**[Reply to this comment]**

We have now included the oxygen generation rate and consumption rate to support our claim (shown in the reply to R3-3). As you pointed out, the oxygen level in the suits significantly changed (Fig. 3C in revised manuscript). However, it remained in the range where insects can survive for days and maintain their locomotor performance. Insects exhibit notable adaptability to hypoxia as well as hyperoxia, which insects are unlikely to encounter in the wild. For example, various insects can grow in wide range of oxygen level (cockroach[23]: 12% - 31%, moth[24]: 10% - 40%, beetle[25]: 10% - 40%). Honeybee[26] and dragonfly[27] are able to fly in atmosphere containing 10% - 40% $O_2$. The previous study in *Drosophila*[28] showed that 90% of the population was surviving ($LS_{90}$: considered as healthy lifespan) at 5% - 80% $O_2$ level for 10 days and that flies in

hyperoxic condition up to 60% $O_2$ level exhibited the climbing performance comparable to those in normoxic condition for 5 days. Therefore, the oxygen supply system is suitable for cyborg insect at least in hours-order.

**[Changes in manuscript]**

Section: 2.2 In-suit Oxygen Concentration Monitoring and Metabolic Rate

Line 185 – 186 in manuscript.

Line 205 – 212 in manuscript.

Section: 3.7 Measurement of Oxygen Generation Rate and Oxygen Consumption Rate

Line 429 – 440 in manuscript.

Fig. 3A.

[R3-7] The Results section contains a large amount of technical detail that may be better suited for the Methods section. For example, the content in lines 236–278 describing the modelling and printing steps could be condensed or moved for clarity.

**[Reply to this comment]**

Thank you for your suggestion. We have relocated unnecessary technical details from the results section to the methods section and supplementary materials. For example, the process of installing the diving suit has been moved to the Methods section. The detailed 3D printing process has been added to the supplementary materials. The revised results section enhances the clarity and conciseness.

[R3-8] Minner comments:

a. Figures 1 and 4 would benefit from the inclusion of scale bars.

b. The supplementary movie should be clearly referenced in the text.

c. Line 59: Please clarify the "disadvantage compared to insects when size matters."

d. Line 62: Dragonflies are generally classified as aerial insects. Please revise.

e. Line 134: Using km/h for cockroach speed seems unusual; cm/s would be more appropriate.

f. Line 137: The phrase "traditional oxygen generation systems" would benefit from a brief explanation of what those systems are, for comparison.

g. Line 187: Clarify how the "first 5 minutes" of walking velocity was computed --- was it a time average, or sampled at a specific time?

h. Line 215: Please elaborate on how structural efficiency and adaptability were evaluated during modelling.

i. Line 328: The comparison to previously measured air-based movement is unclear. Why was this data not measured as part of the current study? Please clarify or provide the relevant new data.

**[Reply to these comments]**

Thank you for your detailed and thoughtful comments. We appreciate your attention to the finer details, and we will address each point as follows:

a. We have added scale bars to all figures.
b. We have revised the manuscript to clearly reference and avoid ambiguous phrasing in relation to the supplementary movies.
c. The statement was intended to emphasize that, in size-limited or confined environments, conventional robotic systems often face disadvantages compared to insects due to their larger body size and reduced adaptability. We have clarified this in introduction section.
d. Apologies for the mistake in the classification of dragonflies.
e. We will revise the speed units to cm/s or mm/s for better clarity and consistency.

f. We thank the reviewer for the suggestion. By “traditional oxygen generation systems,” we refer to methods in which $MnO_2$ powder is directly added into $H_2O_2$ solution to catalytically generate oxygen. Using such system in a confined 1.6 mL reactor results in rapid decomposition, violent bubbling, and liquid agitation, which disrupts the movement of cockroaches. In contrast, in our design, $MnO_2$ is immobilized onto a sponge. The sponge can absorb and retain the $H_2O_2$ liquid.
g. ‘Walking speed during first five minutes’ refers to the average speed across the first ten electrical stimulations, rather than a continuous time average. In the experiment, electrical stimulation was applied every 30 s, with walking speed recorded within the 3 s following each stimulus. The first 10 stimulations corresponded to approximately 5 minutes of experimental time. Thus, ‘walking speed during the first 5 minutes’ refers to the average walking speed across these 10 stimulations.
h. The evaluation of structural efficiency involves testing flexible shells of varying thicknesses to determine the minimum thickness (approximately 1 mm) that resists tearing under external contact forces, such as those exerted by cockroach legs. This ensures sufficient mechanical strength while minimising weight. Adaptability was evaluated by mounting the shell onto live cockroaches after fabrication, observing their crawling and turning behaviour. The shell design was considered adaptable when cockroaches exhibited unrestricted walking, climbing, and turning without noticeable constraints or abnormal postures, indicating no interference with natural locomotion.
i. In the revised manuscript, we have now included experimental data on land-based locomotion (shown in the reply to R3-2) measured under the same implantation and stimulation conditions as in the underwater experiments.

**[Changes in manuscript]**

Section: 1. Introduction

Line 41 – 43 in manuscript.

Section: 2.1 Integrative Design of Diving Suit for Underwater Survival and Mobility

Line 85 – 87 in manuscript.

Section: 2.1 Integrative Design of Diving Suit for Underwater Survival and Mobility

Line 126 in manuscript.

Section: 2.3 Survivability and Locomotion Test

Line 225 – 233 in manuscript.

Fig. 4B.

Reviewer #4:

I co-reviewed this manuscript with one of the reviewers who provided the listed reports. This is part of the Nature Communications initiative to facilitate training in peer review and to provide appropriate recognition for Early Career Researchers who co-review manuscripts.

[Reply to this comment]

We appreciate the clarification and the reviewer's contribution to the evaluation of our manuscript.

## References:


1. Faudzi, A. A. M., Razif, M. R. M., Endo, G., Nabae, H. & Suzumori, K. Soft-amphibious robot using thin and soft McKibben actuator. in *2017 IEEE International Conference on Advanced Intelligent Mechatronics (AIM)* 981–986 (2017). doi:10.1109/AIM.2017.8014146.
2. Jin, H. *et al.* A starfish robot based on soft and smart modular structure (SMS) actuated by SMA wires. *Bioinspir. Biomim.* **11**, 056012 (2016).
3. Crespi, A. & Ijspeert, A. AmphiBot II: An Amphibious Snake Robot that Crawls and Swims using a Central Pattern Generator. in (2006).
4. Zhang, S. *et al.* AmphiHex-I: Locomotory Performance in Amphibious Environments With Specially Designed Transformable Flipper Legs. *IEEEASME Trans. Mechatron.* **21**, 1720–1731 (2016).
5. Ma, X., Wang, G. & Liu, K. Design and Optimization of a Multimode Amphibious Robot With Propeller-Leg. *IEEE Trans. Robot.* **38**, 3807–3820 (2022).
6. Li, M., Guo, S., Hirata, H. & Ishihara, H. Design and performance evaluation of an amphibious spherical robot. *Robot. Auton. Syst.* **64**, 21–34 (2015).
7. Milana, E. *et al.* EELWORM: a bioinspired multimodal amphibious soft robot. in *2020 3rd IEEE International Conference on Soft Robotics (RoboSoft)* 766–771 (2020). doi:10.1109/RoboSoft48309.2020.9115989.
8. Zhu, P. *et al.* Light-Driven Amphibious Mini Soft Robot Mimicking the Locomotion Gait of Inchworm and Water Strider. *Adv. Intell. Syst.* **6**, 2300466 (2024).
9. Guo, S. *et al.* Modeling and experimental evaluation of an improved amphibious robot

with compact structure. *Robot. Comput.-Integr. Manuf.* **51**, 37–52 (2018).

10. Yu, J. *et al.* On a Bio-inspired Amphibious Robot Capable of Multimodal Motion. *IEEEASME Trans. Mechatron.* **17**, 847–856 (2012).
11. Zhong, B. *et al.* On a CPG-Based Hexapod Robot: AmphiHex-II With Variable Stiffness Legs. *IEEEASME Trans. Mechatron.* **23**, 542–551 (2018).
12. Crespi, A., Karakasiliotis, K., Guignard, A. & Ijspeert, A. J. Salamandra Robotica II: An Amphibious Robot to Study Salamander-Like Swimming and Walking Gaits. *IEEE Trans. Robot.* **29**, 308–320 (2013).
13. Li, L. *et al.* Small Multi-Attitude Soft Amphibious Robot. *IEEE Robot. Autom. Lett.* **9**, 1067–1073 (2024).
14. Lin, Z. *et al.* Mudskipper-inspired amphibious robotic fish enhances locomotion performance by pectoral-caudal fins coordination. *Cell Rep. Phys. Sci.* **4**, (2023).
15. Wang, Y., Pancheri, F., Lueth, T. C. & Sun, Y. DuckyDog: An Erect Amphibious Robot with Variable-Stiffness Legs and Passive Fins. *Adv. Intell. Syst.* **7**, 2500267 (2025).
16. Wang, D. *et al.* Miniature Amphibious Robot Actuated by Rigid-Flexible Hybrid Vibration Modules. *Adv. Sci.* **9**, 2203054 (2022).
17. Yang, Z., He, Y., Xing, H., Dai, H. & Wang, L. Fast Swimming Robot Fish Under Countercurrent, Complex Trajectory, and Heavy Load Environments. *J. Field Robot.* **n/a**,.
18. Chao, X., Hameed, I., Navarro-Alarcon, D. & Jing, X. A Controllable Nonlinear Bistable “Fishtail” Boosting Robotic Swimmer with Excellent Maneuverability and High Energy Efficiency. *Soft Robot.* https://doi.org/10.1089/soro.2024.0038 (2024)

doi:10.1089/soro.2024.0038.

19. Sanchez, C. J. *et al.* Locomotion control of hybrid cockroach robots. *J. R. Soc. Interface* **12**, 20141363 (2015).
20. Li, R., Lin, Q., Kai, K., Nguyen, H. D. & Sato, H. A Navigation Algorithm to Enable Sustainable Control of Insect-Computer Hybrid Robot with Stimulus Signal Regulator and Habituation-Breaking Function. *Soft Robot.* **11**, 473–483 (2024).
21. Uchida, K. & Blumstein, D. T. Habituation or sensitization? Long-term responses of yellow-bellied marmots to human disturbance. *Behav. Ecol.* **32**, 668–678 (2021).
22. Li, R., Lin, Q., Kai, K., Nguyen, H. D. & Sato, H. A Navigation Algorithm to Enable Sustainable Control of Insect-Computer Hybrid Robot with Stimulus Signal Regulator and Habituation-Breaking Function. *Soft Robot.* **11**, 473–483 (2024).
23. VandenBrooks, J. M. *et al.* Impacts of Paleo-Oxygen Levels on the Size, Development, Reproduction, and Tracheal Systems of Blatella germanica. *Evol. Biol.* **39**, 83–93 (2012).
24. Callier, V. & Nijhout, H. F. Control of body size by oxygen supply reveals size-dependent and size-independent mechanisms of molting and metamorphosis. *Proc. Natl. Acad. Sci.* **108**, 14664–14669 (2011).
25. Greenberg, S. & Ar, A. Effects of chronic hypoxia, normoxia and hyperoxia on larval development in the beetle *Tenebrio molitor*. *J. Insect Physiol.* **42**, 991–996 (1996).
26. Joos, B., Lighton, J. R. B., Harrison, J. F., Suarez, R. K. & Roberts, S. P. Effects of Ambient Oxygen Tension on Flight Performance, Metabolism, and Water Loss of the Honeybee. *Physiol. Zool.* **70**, 167–174 (1997).
27. Harrison, J. F. & Lighton, J. R. B. Oxygen-Sensitive Flight Metabolism in the Dragonfly

Erythemis Simplicicollis. *J. Exp. Biol.* **201**, 1739–1744 (1998).

28. Skandalis, D. A., Stuart, J. A. & Tattersall, G. J. Responses of *Drosophila melanogaster* to atypical oxygen atmospheres. *J. Insect Physiol.* **57**, 444–451 (2011).
29. Li, C. *et al.* Terradynamically streamlined shapes in animals and robots enhance traversability through densely cluttered terrain. *Bioinspir. Biomim.* **10**, 046003 (2015).
30. Do, S.-H., Batchelor, B., Lee, H.-K. & Kong, S.-H. Hydrogen peroxide decomposition on manganese oxide (pyrolusite): Kinetics, intermediates, and mechanism. *Chemosphere* **75**, 8–12 (2009).
31. Guo, N. *et al.* Impact of aqueous environments on hydrogen peroxide activation by manganese oxides: Kinetics and the critical role of bicarbonate. *Chemosphere* **324**, 138338 (2023).
32. Chen, Y., Doshi, N., Goldberg, B., Wang, H. & Wood, R. J. Controllable water surface to underwater transition through electrowetting in a hybrid terrestrial-aquatic microrobot. *Nat. Commun.* **9**, 2495 (2018).
33. Xiong, Q., Zhou, X., Li, D., Ambrose, J. W. & Yeow, R. C.-H. An Amphibious Fully-Soft Centimeter-Scale Miniature Crawling Robot Powered by Electrohydraulic Fluid Kinetic Energy. *Adv. Sci.* **11**, 2308033 (2024).
34. Wu, G., Wang, Z., Zhao, J., Cui, F. & Cai, X. A Piezoelectrically Driven Microrobot Using a Novel Monolithic Spatial Parallel Mechanism as Its Hip Joint. *J. Bionic Eng.* **21**, 803–820 (2024).
35. Zheng, Q. *et al.* Leech-Inspired Amphibious Soft Robot Driven by High-Voltage Triboelectricity. *Adv. Mater.* **37**, 2417380 (2025).

36. Erickson, J. C., Herrera, M., Bustamante, M., Shingiro, A. & Bowen, T. Effective Stimulus Parameters for Directed Locomotion in Madagascar Hissing Cockroach Biobot. *PLOS ONE* **10**, e0134348 (2015).

37. Xing, H. *et al.* A Novel Small-scale Turtle-inspired Amphibious Spherical Robot. in *2019 IEEE/RSJ International Conference on Intelligent Robots and Systems (IROS)* 1702–1707 (2019). doi:10.1109/IROS40897.2019.8968304.

38. Xiong, Q., Zhou, X., Li, D., Ambrose, J. W. & Yeow, R. C.-H. An Amphibious Fully-Soft Centimeter-Scale Miniature Crawling Robot Powered by Electrohydraulic Fluid Kinetic Energy. *Adv. Sci.* **11**, 2308033 (2024).

## Certificate of Professional Proofreading Service

# EDITORIAL CERTIFICATE

**Date:** Nov 11, 2025

**Manuscript Author(s):** 裕崇 佐藤

**Manuscript Title:** Underwater Suit-Wearing Cyborg Insect Capable of Hours-Long Diving and Terra-Aqua Travel

**To Whom It May Concern:**

This letter confirms that the manuscript corresponding to the information detailed above was edited by a professional, native English-speaking editor at Wordvice.

We guarantee 100% language accuracy in the text, as edited and delivered to the author(s) on the date below. We make no claims as to the substantive matter covered by the paper and have not altered the intent or research content drafted by the author(s).

The author(s) may accept or reject any of our comments or suggestions upon receipt of the document we edited. Should you have any questions or concerns, please contact Wordvice at edit@wordvice.com

Sincerely,
Wordvice

Wordvice is a premier international English editing service. Our mission is to help researchers, scholars, students, and professionals reach their full potential through clear communication in their writing. By providing premium English editing services at affordable rates to clients from around the world, Wordvice seeks to tear down language barriers and contribute to the advancement of research and education.

Signature

Kevin Heintz
Managing Editor, Wordvice

Date of Issue
Nov 11, 2025


Contact Wordvice

| KOREA | U.S. | JAPAN | TAIWAN | CHINA | TURKEY |
|---|---|---|---|---|---|
| edit@essayreview.co.kr | edit@wordvice.com | edit@wordvice.jp | edit@wordvice.com.tw | edit@wordvice.cn | edit@wordvice.com.tr |
| +82 2-1522-9180 | www.wordvice.com | +81 3-4500-8427 | +886 2-7738-1133 | +86 400-820-0510 | +90 0530 390 6216 |
| www.essayreview.co.kr | | www.wordvice.jp | www.wordvice.com.tw | www.wordvice.cn | www.wordvice.com.tr |

# RESPONSES to REVIEWERS

Manuscript ID: NCOMMS-25-24554B

Journal: Nature Communications

Dear Reviewers,

We sincerely thank you for your thoughtful, detailed, and constructive comments. In response, we have performed additional experiments and revised the manuscript accordingly.

A point-by-point response to all reviewer comments is provided on the following pages. **The original comments are presented in black font**, and **our responses are in blue font**. For ease of reference, all modifications made to **the revised manuscript are also highlighted in blue font**.

Sincerely,

All the authors represented by

Hirotaka Sato, Nanyang Technological University, Singapore

## REVIEWER COMMENTS

**Reviewer #2:**

[R2-1] The authors have made substantial efforts to address the concerns raised in the initial review, with significant additions of experimental data, clarifications, and textual improvements. The revisions have greatly enhanced the rigor and clarity of the work. The authors have added critical quantitative data, such as oxygen level monitoring, survival comparisons, and performance benchmarks against existing amphibious robots. The hypothesis-driven restructuring of the introduction and the professional language editing also improve the manuscript's impact. While minor issues remain, the study now demonstrates novelty, methodological robustness, and potential applications in extreme environments, meeting the high standards of Nature Communications. While the revisions are commendable, the following points should be addressed in a minor revision.

**[Reply to this comment]**

Thank you very much for your advice. We are grateful for the positive comments regarding the improvements of the manuscript. We have carefully addressed the remaining minor comments as detailed below, and we believe these revisions further strengthen the manuscript.

[R2-2] The authors note that the system lacks real-time oxygen regulation to match metabolic demands during activity states. Although insects tolerate oxygen fluctuations, the manuscript should discuss this limitation and propose feedback-controlled systems as future work.

**[Reply to this comment]**

In the previous manuscript, we did not explicitly discuss the limitation of the current oxygen generation system in terms of real-time regulation. The oxygen generator does

not dynamically adjust oxygen delivery in response to the insect's varying metabolic demands during different activity states. For future work, active feedback control could be achieved by integrating micro-valves and miniaturized oxygen concentration sensors inside the diving suit. For example, miniaturized oxygen concentration sensors could monitor the oxygen level inside the suit, and micro-valves could modulate the flow of $H_2O_2$ liquid onto the $MnO_2$-sponge. This would allow the reaction to pause when oxygen levels reach an optimal threshold and resume when levels drop below the target concentration, thereby maintaining near-physiological oxygen levels for the insect. This approach would prevent excessive oxygen fluctuations within the suit and avoid exposing the cockroach to suboptimal concentrations for respiration. We have added a brief discussion in the revised manuscript outlining the potential for active regulation strategies in future iterations of the system. Thank you for the advice.

**[Changes in manuscript]**

The following part was added to the Section 2.1.

*Line 122 – 127 in manuscript.*

*Future improvements in oxygen generator could focus on actively regulating oxygen generation rates. For example, integrating miniature oxygen concentration sensors and micropumps would enable quantitative delivery of $H_2O_2$ based on real-time oxygen levels within the suit, thereby achieving dynamic oxygen supply matched to the insect's activity states and overcoming limitations of the current passive system.*

[R2-3] While reagent confinement minimizes risks, data on long-term exposure to byproducts are indirect. Adding a sentence on post-experiment insect recovery would strengthen safety claims.

**[Reply to this comment]**

Thank you for your valuable comment. To further support the safety of our system, we conducted a post-experiment monitoring of five cockroaches exposed to $H_2O_2$ liquid, $MnO_2$ powder and their byproduct during the experiments. Each insect was placed individually in a separate container (19 × 12.5 × 7.5 cm) and observed continuously for three days. All five insects survived without any observable behavioural abnormalities or delayed mortality. These observations provide evidence that hours-long exposure to the reagents and byproducts under our experimental conditions does not significantly affect insect health. We have added a sentence summarizing these post-experiment recovery observations in the revised manuscript.

**[Changes in manuscript]**

The following part was added to the Section 2.1.

*Line 110 – 113 in manuscript.*

*To further evaluate potential biological impact on long-term exposure to byproducts of $H_2O_2$ decomposition, five diving suit wearing cyborg insects were monitored for three days following experimental exposure. All individuals survived throughout the observation period with normal behaviours.*

[R2-4] Success rates in simulated environments are described anecdotally. Including quantitative metrics would bolster robustness assertions.

**[Reply to this comment]**

To provide quantitative evidence of robustness, we have conducted more trials and added the success rate of the tunnel traversal experiments in the revised manuscript. Specifically, three independent trials were conducted under identical $CO_2$–water tunnel conditions, and all cyborg insects equipped with the diving suit successfully traversed both hazardous zones (3/3 successful trials). Thank you for the comment.

**[Changes in manuscript]**

The following part was added to the Section 2.4.

*Line 318 – 325 in manuscript.*

*In contrast, the cyborg insect equipped with the diving suit maintained a regular gait and successfully traversed the entire tunnel (Fig. 5D, Supplementary Movie S3). Three independent trials were conducted under identical $CO_2$–water tunnel conditions, and all cyborg insects wearing the diving suit successfully traversed both hazardous zones (3/3 successful trials). Locomotor activity was preserved throughout both sections, demonstrating that the diving suit enables sustained respiration and movement under combined $CO_2$ exposure and water submersion by preventing gas and water ingress while supplying oxygen independently.*

**Reviewer #5:**

[R5-1] After reviewing the revised manuscript and the authors' detailed responses to the previous round of reviews, I am pleased to see that the authors have undertaken a comprehensive revision of the manuscript. They have effectively rewritten most of the content and conducted extensive supplementary experiments to address concerns regarding safety, system characterization, and comparative analysis. The manuscript is now technically sound and makes a clear contribution to the field of biohybrid robotics. However, the current manuscript lacks a comprehensive examination of limitations and a corresponding discussion. Specifically, I have noted the following points

**[Reply to this comment]**

Thank you for your time reviewing our manuscript and providing insightful comments. In this response, we address your questions point-by-point and have revised the manuscript accordingly.

[R5-2] The authors selected the Madagascar hissing cockroach (*Gromphadorhina portentosa*) as the experimental platform due to its superior characteristics, such as size and load-bearing capacity. However, this species has strong regional specificity and may not be the optimal choice for all potential deployment scenarios. To enhance the practical significance and broader impact of this work, I suggest the authors add a section to the Discussion regarding the potential challenges and coping strategies for developing similar "cyborg suits" for insects with different characteristics (e.g., smaller body sizes or different locomotive habits).

**[Reply to this comment]**

We agree that discussing the challenges and adaptation strategies for extending the proposed diving suit to other terrestrial insects with different morphological and locomotive characteristics would enhance the impact of this work. In addition to *Gromphadorhina portentosa*, several other insects have been explored as cyborg

platforms, including cockroaches (*Blaberus discoidalis*[1], *Periplaneta americana*[2]), locusts (*Locusta migratoria manilensis*[3], *Schistocerca americana*[4]), and beetles (*Mecynorhina torquata*[5], *Zophobas morio*[6]). As terrestrial insects, these species have similar body structure and respiratory system. Specifically, all of them rely on an identical tracheal respiratory system consisting of paired spiracles located on the thoracic and abdominal segments[7–13]. Air enters and exits through these spiracles and is distributed via internal tracheal networks directly to tissues[14]. Because of these similarities, the proposed diving suit concept, which combines an abdominal protective shell with oxygen delivery tubes to the spiracles, could in principle be extended to other terrestrial insect platforms.

However, species-specific differences in morphology, locomotion capacity, and payload capacity introduce several engineering challenges. First, cyborg locusts rely on jumping locomotion. The much higher density of water compared to air would alter the dynamics of this locomotion strategy, make jumping and landing more difficult. The weight of the diving suit would therefore need to be carefully optimized to allow effective jumping while maintaining stable motion in water. Second, some insects, including locusts and beetles, possess wings that fold along the abdomen when not in use. Installing a waterproof shell around the abdomen may interfere with the natural folding and deployment of these wings. Therefore, the suit architecture would require redesign to accommodate wing storage and deployment without mechanical obstruction. Finally, payload capacity varies significantly and limited payload may make locomotion more difficult when additional devices are attached. For example, the relatively small body size of *Zophobas morio* limits its payload capacity and prevent it from carrying sensors required for search-and-rescue applications[15]. Adding a diving suit could further exceed its payload limits and hinder locomotion. In such cases, further miniaturization of the oxygen generation system and the use of ultralight structural materials would be necessary.

Taken together, these factors suggest that while the proposed diving suit concept could

potentially be extended to other terrestrial insect platforms, species-specific morphological and locomotive characteristics will require further optimization in structural design, materials, and device miniaturization to ensure compatibility and functionality. With these engineering adaptations, the diving suit could be applied to a broader range of terrestrial insect, enabling underwater respiration and locomotion across diverse cyborg insect platforms.

**[Changes in manuscript]**

The following part was added to the Section 2.1.

*Line 192 – 208 in manuscript.*

*Moreover, the proposed diving suit concept could be potentially extended to other terrestrial cyborg insect platforms, such as cockroaches*[1,2]*, locusts*[3,4]*, and beetles*[5,6]*. These insects share similar body structures and tracheal respiratory systems*[7–13] *in which oxygen enters through paired spiracles and is distributed through internal tracheal networks*[14]*. These similarities suggest that the strategy of combining an abdominal protective shell with oxygen delivery to the spiracles may also be applicable to terrestrial insect species. However, species-specific differences in morphology, locomotive behaviour, and payload capacity may introduce engineering challenges. For example, jumping insects such as locusts may require optimisation of suit weight and hydrodynamic design, while insects with wings folded along the abdomen may require modified shell geometries to avoid interference with wing deployment. In addition, species with limited payload capacity may necessitate further miniaturisation of the oxygen generation system and the use of ultralight materials. Therefore, adapting the diving suit to different insect species will likely require species-specific optimisation of structural design and device miniaturisation. Such adaptations could further broaden the applicability of the diving suit to diverse terrestrial insects, enabling a wider range of amphibious cyborg insect platforms.*

[R5-3] The current oxygen generation system operates passively; once the $H_2O_2$ is introduced to the $MnO_2$-deposited sponge, the reaction proceeds until the reagents are exhausted. While effective for the demonstrated duration, the manuscript would benefit from a brief discussion on future possibilities for active regulation. For instance, is it theoretically possible to incorporate micro-valves or separation mechanisms to control the reaction rate dynamically?

**[Reply to this comment]**

We thank the reviewer for this insightful suggestion regarding active regulation of oxygen generation. The current system operates in a passive manner: The $H_2O_2$ solution is introduced to the $MnO_2$-deposited sponge at once, and the catalytic decomposition proceeds until the reactants are consumed. This design was intentionally chosen to minimize additional weight, power consumption, and mechanical complexity.

Nevertheless, active regulation of the reaction rate would further enhance control over the oxygen generation process. Such regulation could be achieved through controlled reagent delivery, specifically by modulating the rate at which $H_2O_2$ is introduced to the $MnO_2$ catalyst. For example, integrating microvalves, micropumps, or fluidic gating mechanisms with current oxygen generator could allow stepwise or continuous dosing of $H_2O_2$ onto the catalyst surface, thereby directly controlling the oxygen generation rate. In principle, the dosing rate could be further adjusted according to the oxygen consumption rate of the cockroaches to better match metabolic demand. Moreover, a closed-loop feedback strategy could be implemented in future designs. By incorporating an oxygen sensor within the suit to monitor the internal $O_2$ concentration in real time, the system could determine whether additional $H_2O_2$ dosing is required and accordingly trigger controlled release. This feedback-regulated approach would enable adaptive oxygen supply tailored to metabolic demand and environmental conditions. We have added a brief discussion in the revised manuscript outlining the potential for active regulation strategies in future iterations of the system.

**[Changes in manuscript]**

The following part was added to the Section 2.1.

*Line 122 – 127 in manuscript.*

*Future improvements in oxygen generator could focus on actively regulating oxygen generation rates. For example, integrating miniature oxygen concentration sensors and micropumps would enable quantitative delivery of* $H_2O_2$ *based on real-time oxygen levels within the suit, thereby achieving dynamic oxygen supply matched to the insect's activity states and overcoming limitations of the current passive system.*

[R5-4] The methods describe sealing the flexible shell to the cockroach's first abdominal segment using a nitrile rubber membrane and adhesive to ensure waterproofing. Biologically, this permanent attachment would likely interfere with the insect’s natural molting process. While the focus of this study is appropriately on "hours-long" missions, it would be transparent to include a brief sentence in the discussion acknowledging this constraint. Clarifying that this system is intended for terminal deployment durations or inter-molt periods would improve the biological rigor of the study.

**[Reply to this comment]**

We thank the reviewer for raising this important biological consideration. Insects undergo successive natural molts during development. Cockroaches, as hemimetabolous insects, develop through three stages：egg, nymph, and adult[16]. During the nymphal stage, individuals undergo multiple molts before reaching the final adult form[17,18]. After this succession of molts, the insect attains its terminal adult stage, and no further molting occurs[16]. In the present study, all cockroaches used in the experiments were adult cockroaches. Therefore, potential interference with subsequent

molting is not expected to arise under our experimental conditions.

In addition, the nitrile rubber membrane and adhesive sealing are not intended to permanently integrate with the exoskeleton. The attachment is externally applied and can be mechanically removed if necessary, without structural integration into the cuticle.

To improve clarity, we have added a sentence to state that the system is designed for use in adult insects and for mission durations within their post-molt adult stage.

**[Changes in manuscript]**

The following part was added to the Section 3.2.

*Line 360 –361 in manuscript.*

*G.portentosa (adult, average weight: 5.5 ± 1.0 g, average length: 6.0 ± 0.5 cm) were used in this study.*

The following part was added to the Section 3.4.

*Line 401 – 403 in manuscript.*

*After experiments, the shell can be removed and the membrane can be gently polished off to minimise any restrictions on the cockroach's normal behaviour and daily activities.*

Lastly, we sincerely thank all the reviewers and the editor for their thoughtful suggestions and advice. Their careful comments have greatly improved the manuscript, making the revised version much clearer and more convincing than the original one. We hope that the idea of equipping insects with specialized suits to expand their operational environments will stimulate further discussion and development in this field. We truly appreciate the reviewers' efforts in helping us strengthen this work, and we have learned a great deal from this review process, which will guide our future research.

Sincerely,

All the authors represented by
Hirotaka Sato, Nanyang Technological University, Singapore
hirosato@ntu.edu.sg / +65 6790 5010

## References


1. Sanchez, C. J. *et al.* Locomotion control of hybrid cockroach robots. *J. R. Soc. Interface* **12**, 20141363 (2015).
2. Holzer, R. & Shimoyama, I. Locomotion control of a bio-robotic system via electric stimulation. *Proc 1997 IEEERSJ Int Conf Intell. Robot Syst. Innov. Robot. Real-World Appl.* (1997).
3. Ma, S., Liu, P., Liu, S., Li, Y. & Li, B. Launching of a Cyborg Locust via Co-Contraction Control of Hindleg Muscles. *IEEE Trans. Robot.* **38**, 2208–2219 (2022).
4. Giampalmo, S. L. *et al.* Generation of complex motor patterns in american grasshopper via current-controlled thoracic electrical interfacing. in *2011 Annual International Conference of the IEEE Engineering in Medicine and Biology Society* 1275–1278 (2011). doi:10.1109/IEMBS.2011.6090300.
5. Cao, F. & Sato, H. Insect–Computer Hybrid Robot Achieves a Walking Gait Rarely Seen in Nature by Replacing the Anisotropic Natural Leg Spines With Isotropic Artificial Leg Spines. *IEEE Trans. Robot.* **35**, 1034–1038 (2019).
6. Fitzgerald, L. *et al.* Zoborg: On-Demand Climbing Control for Cyborg Beetles. *Adv. Sci.* **12**, e02095 (2025).
7. Pendar, H., Kenny, M. C. & Socha, J. J. Tracheal compression in pupae of the beetle Zophobas morio. *Biol. Lett.* **11**, 20150259 (2015).
8. Wagner, J. M. *et al.* Isometric spiracular scaling in scarab beetles—implications for diffusive and advective oxygen transport. *eLife* **11**, e82129 (2022).
9. Harrison, J. F., Lafreniere, J. J. & Greenlee, K. J. Ontogeny of tracheal dimensions and

gas exchange capacities in the grasshopper, *Schistocerca americana*. *Comp. Biochem. Physiol. A. Mol. Integr. Physiol.* **141**, 372–380 (2005).

10. Weis-Fogh, T. Respiration and Tracheal Ventilation in Locusts and other Flying Insects. *J. Exp. Biol.* **47**, 561–587 (1967).
11. Woodman, J. D., Cooper, P. D. & Haritos, V. S. Neural regulation of discontinuous gas exchange in *Periplaneta americana*. *J. Insect Physiol.* **54**, 472–480 (2008).
12. Case, J. F. The median nerves and cockroach spiracular function. *J. Insect Physiol.* **1**, 85–94 (1957).
13. Kaars, C. Innervation and control of tension in abdominal muscles of *Blaberus discoidalis* and *Gromphadorhina portentosa*. *J. Insect Physiol.* **29**, 371–376 (1983).
14. Khan, S. & Staples, A. E. Mechanisms of insect respiration. *Nat. Rev. Phys.* **7**, 135–148 (2025).
15. Fraga, C. J., Brown, M. S. & Xu, N. W. Advances in Invertebrate Biohybrid Robotics: Leveraging Nature for Locomotion and Sensing in Engineered Systems. *Adv. Intell. Syst.* **7**, 2401105 (2025).
16. Kaleka, A. S. *et al.* Larval Development and Molting. in *Edible Insects* (IntechOpen, 2019). doi:10.5772/intechopen.85530.
17. Kunkel, J. G. Cockroach Molting. I. Temporal Organization of Events during Molting Cycle of Blattella germanica (L.). *Biol. Bull.* **148**, 259–273 (1975).
18. Kunkel, J. G. Cockroach Molting. II. The Nature of Regeneration-Induced Delay of Molting Hormone Secretion. *Biol. Bull.* **153**, 145–162 (1977).
